# LRG1: an emerging player in disease pathogenesis

**DOI:** 10.1186/s12929-022-00790-6

**Published:** 2022-01-21

**Authors:** Carlotta Camilli, Alexandra E. Hoeh, Giulia De Rossi, Stephen E. Moss, John Greenwood

**Affiliations:** Institute of Ophthalmology, University College London, London, UK

**Keywords:** LRG1, Inflammation, Immunity, Neovascularization, Vascular normalization, Fibrosis, Cancer, Diabetes, Endothelial cell, Neutrophils

## Abstract

The secreted glycoprotein leucine-rich α-2 glycoprotein 1 (LRG1) was first described as a key player in pathogenic ocular neovascularization almost a decade ago. Since then, an increasing number of publications have reported the involvement of LRG1 in multiple human conditions including cancer, diabetes, cardiovascular disease, neurological disease, and inflammatory disorders. The purpose of this review is to provide, for the first time, a comprehensive overview of the LRG1 literature considering its role in health and disease. Although LRG1 is constitutively expressed by hepatocytes and neutrophils, *Lrg1*^*−/−*^ mice show no overt phenotypic abnormality suggesting that LRG1 is essentially redundant in development and homeostasis. However, emerging data are challenging this view by suggesting a novel role for LRG1 in innate immunity and preservation of tissue integrity. While our understanding of beneficial LRG1 functions in physiology remains limited, a consistent body of evidence shows that, in response to various inflammatory stimuli, LRG1 expression is induced and directly contributes to disease pathogenesis. Its potential role as a biomarker for the diagnosis, prognosis and monitoring of multiple conditions is widely discussed while dissecting the mechanisms underlying LRG1 pathogenic functions. Emphasis is given to the role that LRG1 plays as a vasculopathic factor where it disrupts the cellular interactions normally required for the formation and maintenance of mature vessels, thereby indirectly contributing to the establishment of a highly hypoxic and immunosuppressive microenvironment. In addition, LRG1 has also been reported to affect other cell types (including epithelial, immune, mesenchymal and cancer cells) mostly by modulating the TGFβ signalling pathway in a context-dependent manner. Crucially, animal studies have shown that LRG1 inhibition, through gene deletion or a function-blocking antibody, is sufficient to attenuate disease progression. In view of this, and taking into consideration its role as an upstream modifier of TGFβ signalling, LRG1 is suggested as a potentially important therapeutic target. While further investigations are needed to fill gaps in our current understanding of LRG1 function, the studies reviewed here confirm LRG1 as a pleiotropic and pathogenic signalling molecule providing a strong rationale for its use in the clinic as a biomarker and therapeutic target.

## Background

**Fig. 1:**
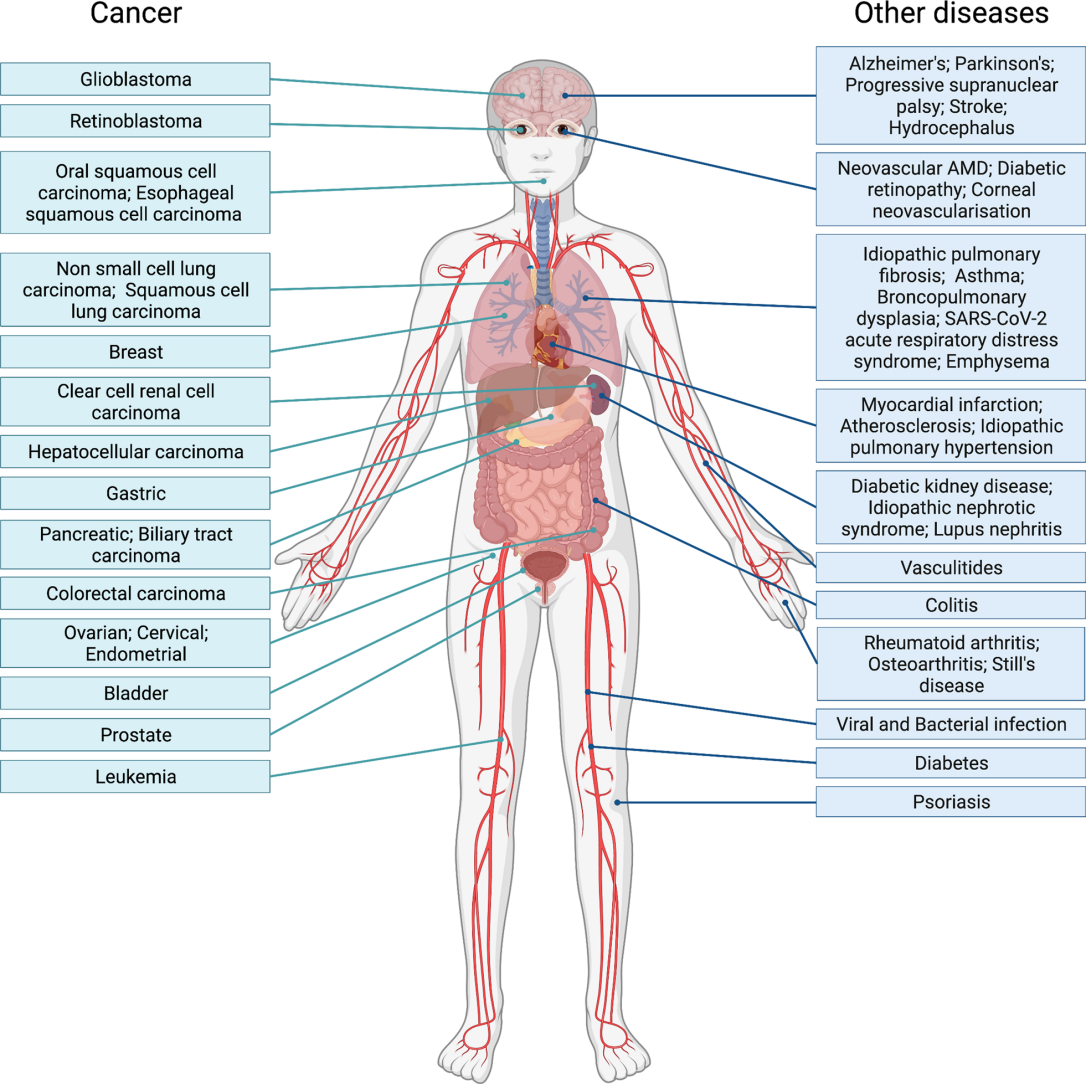
LRG1 expression in disease. Human diseases characterized by the upregulation of LRG1 expression. These illustrate the pleiotropic and wide-spanning role LRG1 plays in disease. Considerable data now show that LRG1 is a contributing factor to the disease process and not simply a response to the condition

Leucine-rich α-2 glycoprotein 1 (LRG1) is a secreted member of the family of leucine-rich repeat (LRR) proteins and was first discovered in human serum in 1977 [1]. The LRR motifs are evolutionarily conserved and have been found in plants, animals, bacteria, and fungi. Many are involved in protein–protein interactions and, among various other functions, serve as pattern recognition motifs for the innate immune system [2, 3]. Although discovered decades ago, little is still known about the role of LRG1 under physiological conditions as *Lrg1*^*−/−*^ mice show no overt phenotypic abnormality. However, interest in this molecule has grown considerably in recent years as evidence accumulates for its contribution to a wide range of human diseases (Fig. 1). LRG1 is a multifunctional pathogenic signalling molecule which, amongst other activities, modulates the TGFβ pathway in a highly context-dependent manner. LRG1 was first described as an important player in pathological angiogenesis [4] but, since then, evidence for a much wider range of biological functions has accumulated, as discussed in this review. A substantial increase in LRG1 expression has been reported in cancer and diabetes, both responsible for a great burden of morbidity and mortality worldwide, but also in infections, cardiovascular, kidney, lung, neurological and autoimmune disorders. Underlying many, but not all, of the pathogenic contributions LRG1 makes in these diseases are its effects on the vasculature, and these will be discussed in detail. Moreover, whilst correlation of LRG1 levels with disease does not imply causation, there is strong evidence that elevated or ectopic expression directly leads to disease pathology.

As a role for LRG1 in disease gains traction, there is now considerable interest in targeting its activity therapeutically. Accordingly, the recent development of a function-blocking antibody [5] might provide researchers with tools to counteract aberrant LRG1 biological activities in a wide range of pathological settings [6]. Here, we provide an overview of the LRG1 literature considering its role as a contributing factor in disease and discussing its potential clinical application as a novel biomarker and therapeutic target.

## Review

### LRG1 molecular structure

**Fig. 2:**
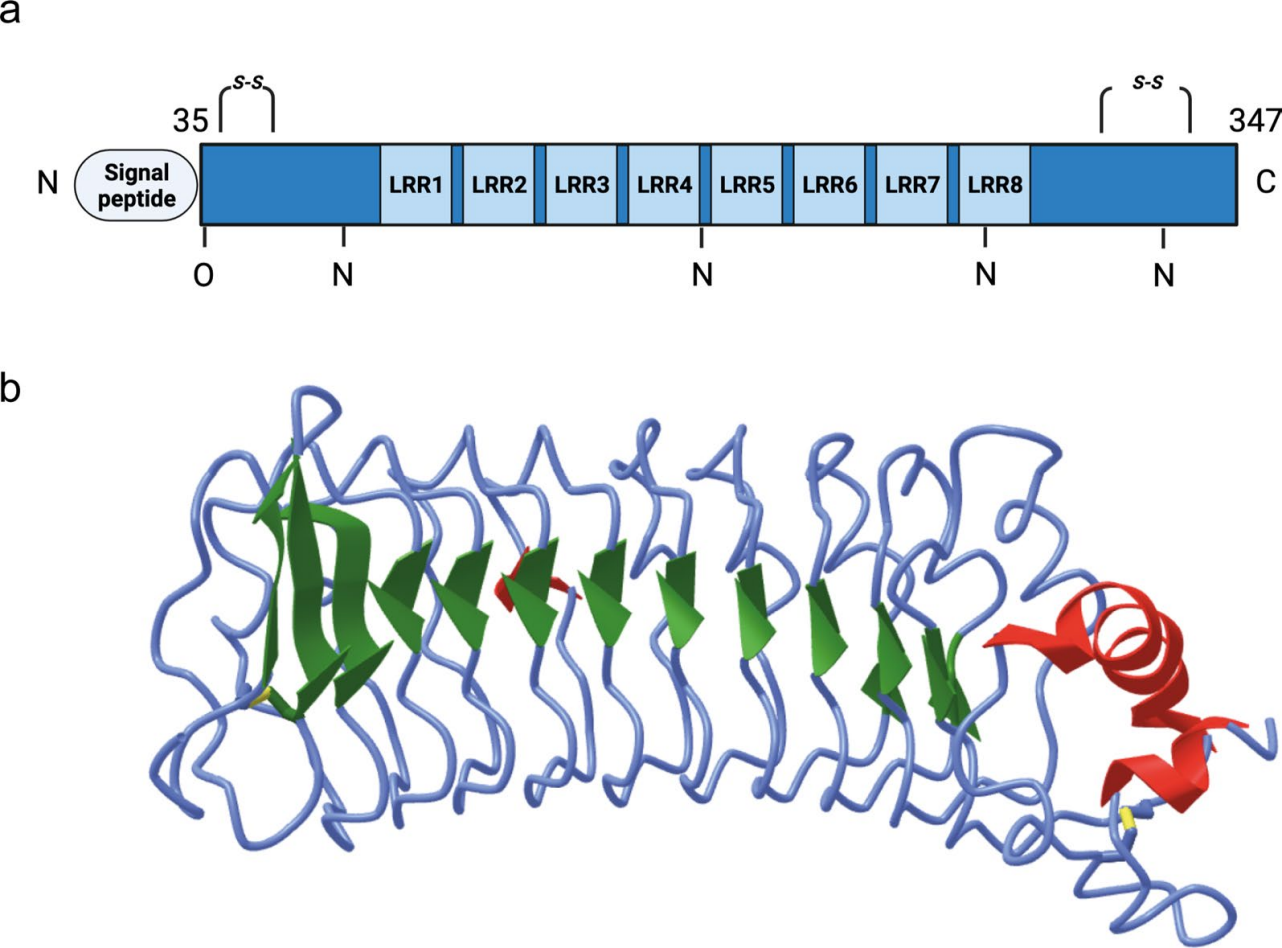
LRG1 protein structure. Schematic representation of LRG1 protein structure. **A** LRG1 is a 312 aa protein which contains 8 leucine-rich repeats (LRR), 4 N-linked, 1 O-linked glycosylation sites and 2 disulphide bonds. Upon cleavage of the N-terminal signal peptide, LRG1 is released in the extracellular space. The mature form, around 50 kDa, may vary in weight depending on the glycosylation pattern and multimer formation. **B** LRG1 structure as predicted by ALPHAFOLD2 through deep learning algorithms [223]. β-sheet in green, α-helix in red

LRG1 was first isolated from human serum in 1977 [1] and its amino acid sequence was determined in 1985 [7]. It consists of a single polypeptide chain of 312 amino acid residues and contains 8 LRRs (Fig. 2). LRRs are protein–ligand interaction motifs, typically arranged in repetitive stretches of variable length. Each LRR consists of 19–29 amino acids, comprising a well-conserved N-terminal stretch of 9–12 amino acids, which is rich in the hydrophobic amino acid leucine, and a C-terminal domain that varies in length, sequence, and structure. Multiple repeats are typically arranged together to form a horseshoe shaped solenoid protein domain with a concave surface providing a platform for protein–protein interactions [8] (Fig. 2). The negatively charged leucine-rich N-terminal stretches of the repeats form β-strands located towards the inside of the horseshoe shaped domain [9] and represent ideal binding sites for cationic proteins such as TGFβ [8]. Although its crystal structure has not yet been reported, LRG1 has been predicted to contain a leucine-rich C-terminal domain (LRC) connected to the LRRs by several loops [4].

LRG1 is a glycoprotein with a carbohydrate content of 23% [1] and predicted to contain 5 glycosylation sites [8] (uniprot.org) (Fig. 2). Indeed, several authors have shown that the exact molecular weight of LRG1 varies due to differences in glycosylation [10, 11]. Deglycosylated LRG1 has a molecular weight of about 34–36 kDa, whereas glycosylated LRG1 can reach up to 55–60 kDa. It has been shown that neutrophil-derived LRG1 is glycosylated differently from serum LRG1 [10], and that CD11b^pos^ F4/80^neg^ neutrophils express LRG1 in more different molecular sizes than CD11b^pos^ F4/80^pos^ macrophages [11]. It is not known how the glycosylation or deglycosylation of LRG1 is regulated in vivo, nor what impact differential glycosylation patterns may have on function. However, LRG1 from serum samples of pancreatic [12] and colorectal cancer patients [13] shows aberrant glycosylation patterns with regards to content of mannose, fucose and sialic acid suggesting that alterations in sugar chains may influence LRG1 function in cancer.

### LRG1 physiological tissue expression

**Fig. 3:**
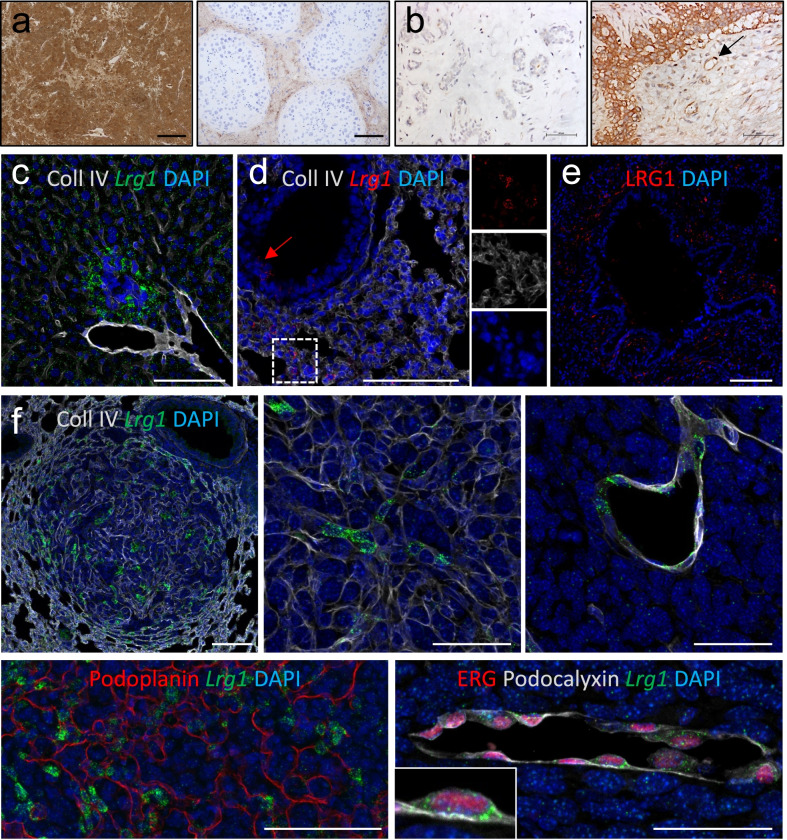
LRG1 expression in normal and cancer tissues. **A** A polyclonal (Proteintech) or monoclonal (Magacizumab) antibody was used for the detection of human LRG1 (brown) respectively in human liver (left, scale bar: 250 µm) and testis (right, scale bar: 100 µm). **B** Immunohistochemistry showing upregulation of LRG1 expression (brown) in human breast cancer (right) compared to healthy control (scale bar: 60–62 µm). The arrow indicates an example of LRG1^pos^ blood vessel. **C** *Lrg1* mRNA (green) detected by RNA scope and immunofluorescence for Collagen IV (white) profiling tissue vessels in normal mouse liver (scale bar: 100 µm). **D** *Lrg1* mRNA (red) detected by RNA scope and immunofluorescence for Collagen IV (white) profiling tissue vessels in normal mouse lung (scale bar: 100 µm). The arrow points to *Lrg1* mRNA expressed by lung alveolar epithelium while the box shows details of additional LRG1^pos^ stromal cells. **E** An anti-human polyclonal antibody (Proteintech) was used for the detection of LRG1 (red) in normal human lung (scale bar: 100 µm). **F** Upregulation of LRG1 expression in murine metastatic lung tumours. Top: examples of *Lrg1* mRNA detected by RNA scope (green) and Collagen IV stained by immunofluorescence (white); left: low magnification of metastatic tumour mass (scale bar: 100 µm); middle: *Lrg1* expression by cancer cells or cancer-associated fibroblasts (scale bar: 50 µm); right: *Lrg1* expression by tumour vessels (scale bar: 50 µm). Bottom left: *Lrg1* mRNA (green) detected by RNA scope and immunofluorescence for Podoplanin (red) (scale bar: 100 µm); right: *Lrg1* mRNA detected by RNA scope (green) and immunofluorescence for the endothelial markers ERG (red) and Podocalyxin (white) (scale bar: 50 µm). Murine metastatic tumour samples were kindly provided by M. Singhal and H. G. Augustine, Heidelberg University, Germany. Human samples were purchased from Biomax and Covance.

Under physiological conditions, LRG1 is primarily synthesized by hepatocytes (Fig. 3A left, C) and neutrophils [14] (Fig. 5), although marginal expression levels have also been reported in lung (Fig. 3D, E), kidney, heart, skin, brain, and testis. The majority of LRG1 appears to be expressed as a monomer, but other higher molecular weight multimers may also be secreted. Histological studies are partly confounded by the poor reliability of available antibodies and the blood-borne nature of LRG1 resulting in diffuse extracellular staining in organs with limited vascular exclusion. However, in a testis cross section, where seminiferous tubules are isolated from the surrounding interstitial space by the Sertoli cell barrier, LRG1 appears to localize exclusively in the extracellular matrix (ECM), where it is likely sequestered following diffusion from the nearby blood vessels (Fig. 3A, right). Immunohistochemistry on tissue sections demonstrated that alveolar epithelial cells [15, 16] (Fig. 3D), renal tubular epithelial cells [17] and interstitial cells [18] express LRG1 in the lung, kidney and heart respectively, while cell-specific loss of function in vivo experiments showed that fibroblasts may represent a key source of LRG1 in the normal skin [19]. *Lrg1* also belongs to a cluster of genes upregulated in adipose tissue during late embryonic and early postnatal development, at the time when adipocytes start accumulating lipids [20]. Recent studies revealed not only that LRG1 is indeed secreted by white and brown adipocytes [21, 22] but also that, whereas *Lrg1* is similarly transcribed in the liver and adipose tissue, its protein levels are significantly higher in the latter [22]. On the other hand, endothelial cells appear to express either undetectable or low levels of LRG1 at multiple sites. For example, independent studies reported LRG1 expression in kidney endothelial cells using either in situ co-hybridization for *Lrg1* and *Cd31* transcripts [23] or immunohistochemistry on laser-captured glomeruli [24], while putative LRG1^pos^ endothelial cells were detected in lung [16] and brain [25] sections by immunohistochemistry, although co-stainings for specific endothelial markers are required to confirm these observations. However, it is worth considering that histological studies involving secreted proteins do not establish with any certainty whether the cell types co-localizing with LRG1 indeed contribute to its production.

To conclude, LRG1 is present in the serum of healthy individuals and may be expressed at the tissue level. Nevertheless, LRG1 physiological function remains poorly understood with knockout mice exhibiting no overt phenotypic abnormality.

### Regulation of *Lrg1* expression

**Fig. 4:**
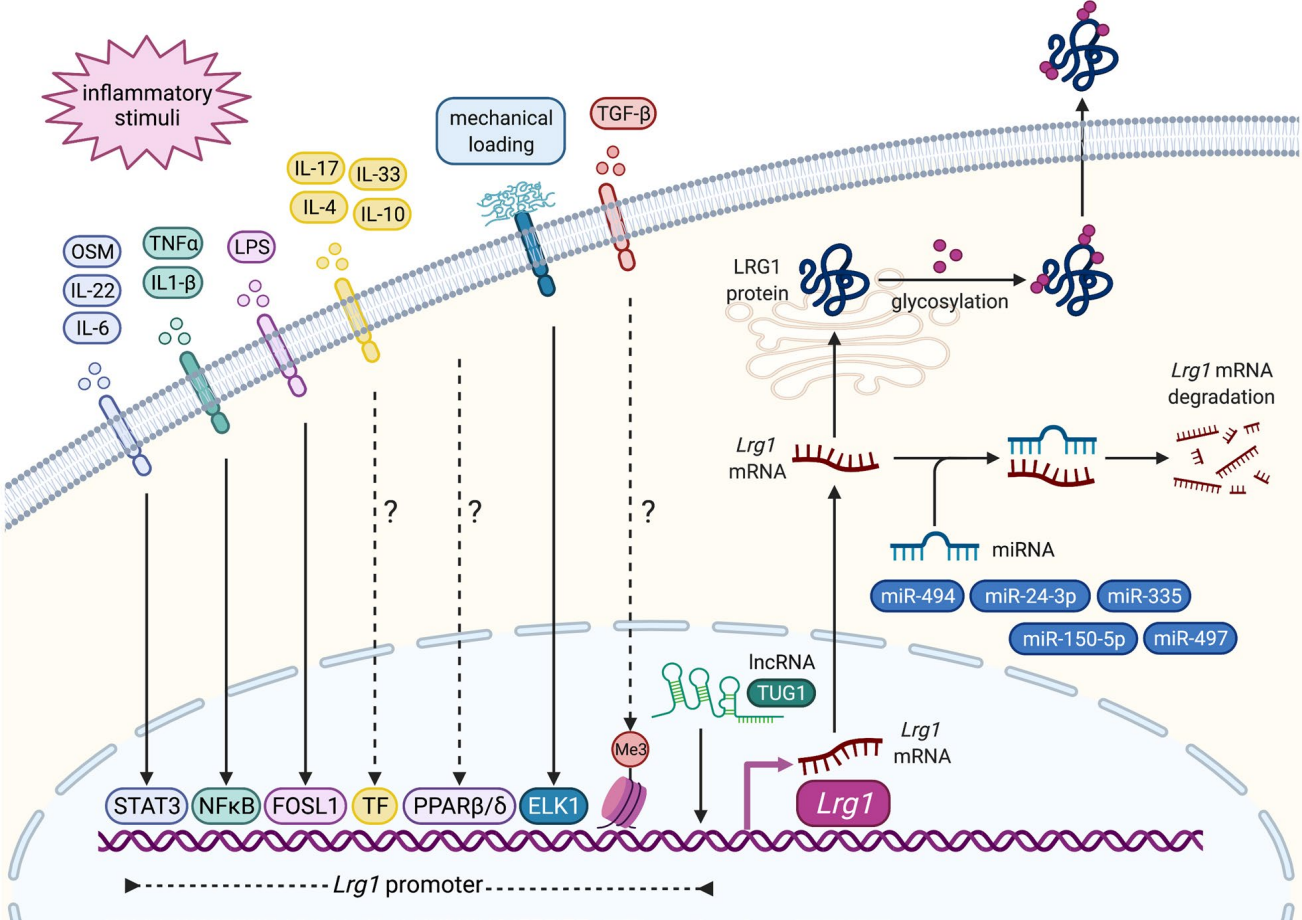
Regulation of *Lrg1* expression. Schematic representation of the mechanisms regulating LRG1 expression at transcriptional and post-transcriptional levels. Several pro-inflammatory signalling molecules, including cytokines and bacteria-derived LPS, drive the expression of *Lrg1* by promoting the activation of different transcription factors in a cell- and context-specific manner. Importantly, the combined stimulation with different cytokines has a synergistic effect on the activity of *Lrg1* promoter. Biomechanical forces also stimulate *Lrg1* expression through the FAK/ERK/ELK1 axis. Various non-coding RNAs have been associated with *Lrg1* regulation. While the lncRNA TUG1 directly facilitates *Lrg1* transcription, miR-335, miR-494, miR-497, miR-150-5p and miR-24-3p promote the degradation of *Lrg1* mRNA and therefore are often downregulated in cancer. TGFβ-induced methylation has also been reported to favour expression of the *Lrg1* gene. Finally, LRG1 protein is differentially glycosylated in a cell- and function-specific fashion prior to secretion into the extracellular space. *OSM* Osteopontin, *lncRNA* long non-coding RNA

Although we lack a comprehensive understanding of how *Lrg1* expression is regulated, the IL-6/STAT3 signalling pathway stands as one of the key drivers of *Lrg1* transcription (Fig. 4). Indeed, conditional knockout of the transcription factor STAT3 in mammary epithelium compromises the expression of *Lrg1* [26]. Furthermore, the observation that *Lrg1* deletion attenuates the IL-6/STAT3 cascade by reducing the expression of IL-6 receptor (IL-6R) in naïve CD4^pos^ lymphocytes [27] implies that LRG1 might represent a downstream modulator of this pathway. Interestingly, STAT3 mediates the transcription of *Lrg1* also upon stimulation with IL-22 [28] and Oncostatin M [29], suggesting that LRG1 might be activated under different inflammatory conditions. Indeed, several in vitro studies described IL-1β, IL-17, TNFα, IL-4, IL-33 and IL-10 as additional LRG1 regulators in hepatoma cells [30], endothelial cells [31], bronchial epithelial cells [15] and “alternatively activated” (M2) macrophages [32] (Fig. 4). Using luciferase-expressing human hepatoma cells, Naka and Fujimoto demonstrated that combinatorial stimulation with IL-6 and IL1-β, which respectively signal through the transcription factors STAT3 and NFkB, has a synergistic effect on the activity of the *Lrg1* promoter, thus confirming the hypothesis that *Lrg1* expression can be boosted by the co-presence of multiple cytokines [30]. Amongst the transcriptional regulators of *Lrg1* expression, in silico and ChIP analysis revealed that PPARβ/δ is recruited to PPAR responsive elements within the regulatory region of the *Lrg1* promoter in human dermal fibroblasts [19]. Additionally, RNA-seq studies recently identified FOS-like 1 as a novel transcription factor induced early on by lipopolysaccharide (LPS) and promoting *Lrg1* expression in mouse lung endothelial cells [33] (Fig. 4). Other than pro-inflammatory cues, it has been reported that mechanical loading derived from the ECM during scar formation induces in dermal fibroblasts the activation of the FAK-ERK signalling pathway and ultimately the expression of *Lrg1* by the transcription factor ELK1 [34] (Fig. 4). Non-coding RNAs and epigenetic changes are also primarily involved in the regulation of gene expression. Interestingly, while the long non-coding RNA TUG1 facilitates the synthesis of LRG1 in ovarian cancer cells [35], TGFβ has been recently shown to induce histone H3 lysine 4 (H3K4) trimethylation and subsequent transcription of *Lrg1* and other genes of the TGFβ superfamily in prostate cancer cells, pointing towards the establishment of a positive feedback loop and supporting the theory that LRG1 modulates the TGFβ axis in cancer [36] (Fig. 4).

*Lrg1* expression can be controlled at the post transcriptional level (Fig. 4). Various microRNAs (miRNAs) have been shown to inhibit *Lrg1* expression through mRNA degradation, including miR-494 [37], miR-24-3p [38], miR-335 [39], miR-497 [40] and miR-150-5p [41]. In particular, miR-335 suppresses neuroblastoma cell migration by inhibiting the expression of *Lrg1* and other genes of the non-canonical TGFβ network including *ROCK1* and *MAPK1*. Given that i) suppression of each target similarly affects the phosphorylation status of the motor protein MLC and that ii) miRNAs are known to target multiple genes within the same genetic pathway, it is reasonable to speculate that LRG1 might alter the cell migratory machinery through upstream modulation of non-canonical TGFβ cascades [39]. Alternatively, following myocardial infarction, miR-494-driven *Lrg1* suppression downregulates the Wnt/β-catenin signalling pathway ultimately promoting cell migration and tissue remodeling, which are key for the restoration of organ functionality [37]. The opposite outcomes of LRG1 inhibition on cell motility described here corroborate the hypothesis that LRG1 exerts the most diverse and conflicting functions through interfering with various signalling pathways, as also exemplified by the different and apparently counterintuitive roles it plays in the heart as described later in this review. Further, it is worth noting that, although the large majority of information related to *Lrg1* post transcriptional regulation comes from studies on cancer and other pathological conditions, it is becoming progressively more evident that this may play an important role also in physiology thus explaining some of the discrepancies in mRNA *versus* protein levels that have been observed, for example, in the liver and adipose tissue [22].

### LRG1 and innate immunity

**Fig. 5:**
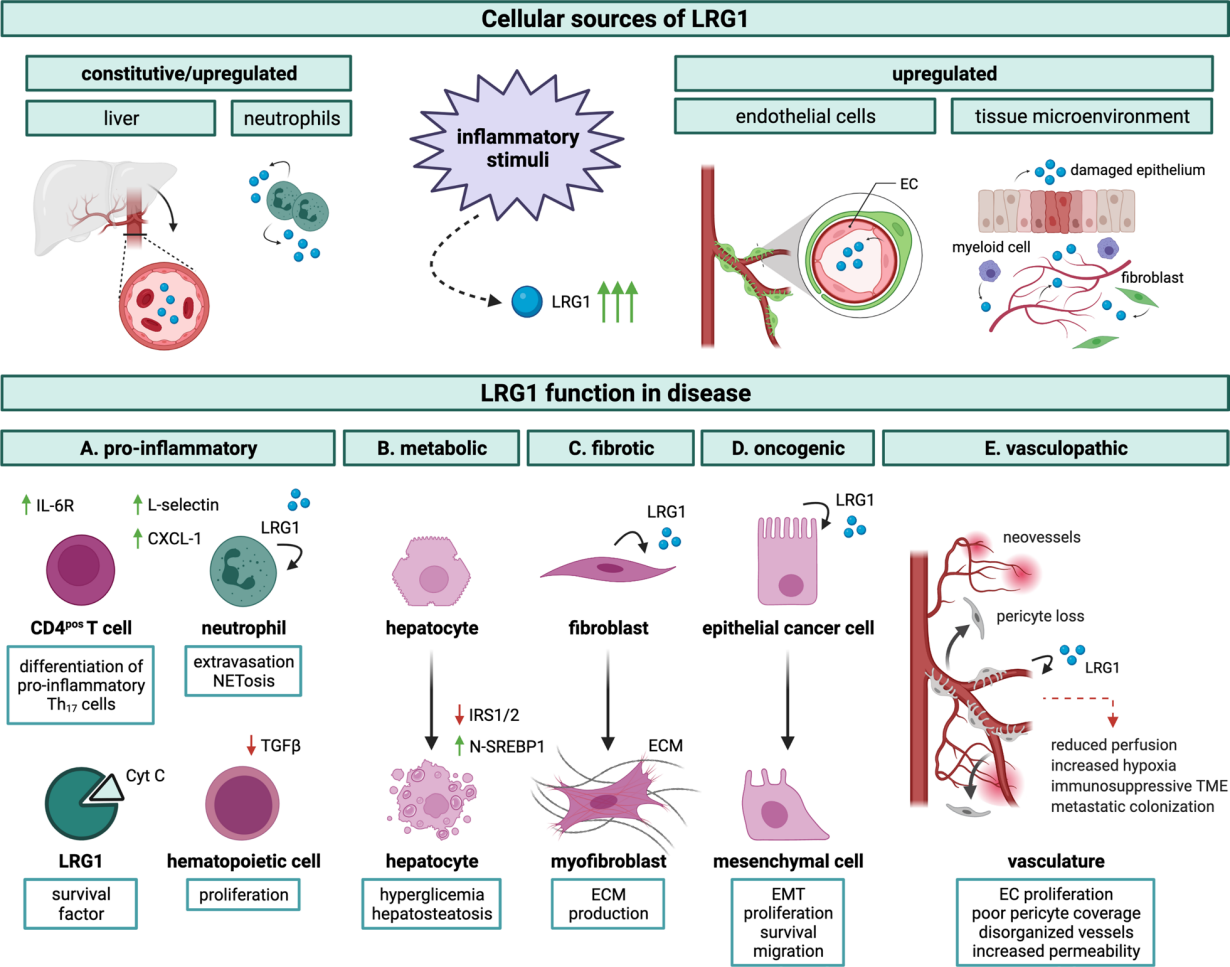
LRG1 functions in disease progression. Schematic representation of LRG1 cell sources and pathological functions. Following various inflammatory stimuli, including infection, injury, autoimmune disease, and tumour-associated inflammation, LRG1 may be produced systemically and/or at the local tissue level. Predominant cellular sources include hepatocytes, neutrophils, and endothelial cells but also other components of the tissue microenvironment, namely epithelial cells, fibroblasts, and other types of myeloid cells. LRG1 pathogenic functions may be initiated through autocrine and paracrine activity and can be broadly classified into **A** pro-inflammatory: LRG1 favours immune cell participation at the inflammatory site by (i) counteracting TGFβ-driven anti-proliferative function on hematopoietic progenitors; (ii) promoting the extravasation and activation of neutrophils; and (iii) enhancing the differentiation of naïve CD4^pos^ T cells into pro-inflammatory Th_17_ lymphocytes. Additionally, LRG1 acts as a survival factor for circulating immune cells by neutralizing Cyt *c* cytotoxicity. **B** metabolic: LRG1 affects hepatocytes by suppressing fatty acid catabolism, promoting lipogenesis through activation of SREBP1, and inhibiting the expression of IRS1/2 thus contributing to hepatosteatosis and hyperglycemia. **C** fibrotic: LRG1 promotes the functional transition of fibroblasts (and epithelial cells, not shown) into ECM-producing cells in fibrosis. **D** oncogenic: LRG1 contributes to cancer cell malignancy by promoting EMT and exerting proliferative and anti-apoptotic functions. **E** vasculopathic: LRG1 affects vessel stability by promoting dysfunctional angiogenesis and interfering with EC-pericyte crosstalk. These effects contribute to the formation of disorganized and highly permeable capillaries. Notably, these outcomes indirectly sustain and amplify some of the direct effects, as dysfunctional and poorly perfused vessels are responsible for the establishment of a highly hypoxic microenvironment which, in turn, contributes to fibrosis, immunosuppression and cancer cell aggressiveness. *Cyt c* cytochrome c, *IRS* insulin receptor substrate, *N-SREBP1* nuclear sterol regulatory element binding protein 1, *ECM* extracellular matrix, *EMT* epithelial-mesenchymal transition, *EC* endothelial cell

One hypothesis that has been postulated regarding the physiological role of LRG1 is that it is an acute phase protein (APP), as levels increase rapidly in the serum following microbial infections and other inflammatory stimuli [42]. The APPs, which are synthesized by the liver in response to several pro-inflammatory cytokines [43], are non-specific innate components responsible for a primitive immune reaction before the activation of acquired immunity. The concentration of circulating APPs increases at least 1000-fold during an inflammatory response, in direct relation to the severity of the disorder [44]. To evaluate whether LRG1 is an acute-phase protein, Shirai and colleagues injected intravenously, into wild-type mice, different doses of LPS, a major membrane component of Gram-negative bacteria known to cause acute inflammatory responses. They reported a dose-dependent enhancement of LRG1 expression in the liver, similar to that observed for other major APPs in the mouse [44]. The beneficial contribution, if any, of LRG1 as an acute phase protein is not entirely clear. One of the most intriguing studies reports that LRG1 strongly binds to cytochrome c (Cyt *c*) [8, 45]. Cyt *c* is located in the mitochondrial intermembrane space but, in response to death signals, translocates into the cytoplasm where it binds to the apoptotic peptidase activating factor Apaf-1. This in turn, following a conformational change, initiates the intrinsic pathway of apoptosis through activation of procaspase-9 [46]. Cyt *c* is also known to be released from apoptotic cells into the extracellular space, promoting further cell death and inflammation in vivo. In 2010, Codina *et al*. reported that LRG1 and Apaf-1 share similar amino acid sequences and, therefore, interact similarly with Cyt *c*. LRG1 added to cultures of human lymphocytes was shown to protect against the toxic effects of Cyt *c* either released from apoptotic cells or experimentally administered [42]. The authors also reported that a substitution of alanine for tri-methyl-lysine at position 72 in Cyt *c* prevents LRG1 binding. Since knock-in mice expressing this variant of Cyt *c* display an extensive reduction in peripheral B and T cells [47], this raises the possibility that LRG1, when bound to Cyt *c*, acts as a survival factor for lymphocytes and possibly other cell types (Fig. 5). Indeed, the high affinity of LRG1 for Cyt *c* would make it a very effective trap to sequester Cyt *c* and protect cells from apoptosis, ultimately favouring their survival [8]. However, it is imperative to note that LRG1 was shown to protect in vitro from Cyt *c* cytotoxicity also in the presence of a vast molar excess of Cyt *c* suggesting that its anti-apoptotic functions are not exclusively mediated by steric hindrance [8]. Moreover, the observation that exogenous LRG1 ameliorates cell viability per se, regardless of Cyt *c* addition [42], demonstrates that it can exert protective functions not only through the clearance of pro-apoptotic factors, such as Cyt *c*, but also via direct modulation of alternative survival pathways, as confirmed by various studies on cancer and discussed later in this review [48–50]. While counteracting the negative effects of systemic inflammation on cell viability, LRG1 has more recently been shown to exert a similar anti-apoptotic function in the cytosol of cancer cells, where it directly competes with Apaf-1 for binding Cyt *c* when mitochondria undergo membrane permeabilization in the absence of a committed death signal [51]. Taking advantage of the binding affinity of LRG1 for Cyt *c*, Weivoda *et al*. developed an indirect enzyme-linked immunosorbent assay for the quantification of human LRG1, in which Cyt *c* is employed as the capturing agent and an anti-LRG1 monoclonal antibody is used to detect the captured target [52].

Other than being produced by the liver and released systemically, LRG1 is also physiologically synthesized by neutrophils. Neutrophils are powerful mediators of innate immunity and differentiate in the bone marrow from myeloid progenitors in response to granulocyte colony stimulating factor (G-CSF) [53]. LRG1 is produced early during G-CSF-induced neutrophilic granulopoiesis and before the characteristic segmented nuclei of neutrophils appear, and persists through the final differentiation stage. This could explain why *Lrg1* expression has been detected also in the progenitor-rich mononuclear cell fraction of the bone marrow but not in peripheral lymphocytes and monocytes [14]. Constitutive expression of LRG1 accelerates neutrophilic granulopoiesis in vitro, suggesting a direct role for LRG1 in myeloid cell differentiation [54]. In human neutrophils, LRG1 is mainly packed with lactoferrin into cytoplasmatic secondary granules but it also co-localizes to a lesser extent with gelatinase-containing tertiary granules. Most importantly, like other granule proteins, LRG1 can be released into the extracellular space upon neutrophil activation at sites of infection or inflammation. Interestingly, although exhibiting a different glycosylation pattern, neutrophil-derived LRG1 retains its affinity for Cyt *c* [10], further highlighting the importance of this still partially defined function. Other than exhibiting antimicrobial and proteolytic activities, some neutrophil-derived proteins have already been shown to regulate the function of other immune cell types [55]. Similarly, LRG1 has been demonstrated to mitigate the anti-proliferative effects of TGFβ on human hematopoietic and myeloid progenitors [10], therefore potentially contributing to the accumulation of immune cells at the tissue level (Fig. 5). A robust body of evidence further supports the hypothesis that LRG1 can modulate the tissue microenvironment by regulating the function of multiple cell types, as discussed extensively in this review. In particular, LRG1 has been reported to affect, at least partly in an autocrine fashion, the function of neutrophils by modulating the formation of neutrophil extracellular traps (NETs), as well as the expression of L-selectin [56] and CXCL-1 [57] which both regulate the adhesion of neutrophils to the endothelium, thus contributing to their recruitment to the site of injury (Fig. 5).

Aside from neutrophils, *Lrg1* has been found to also be part of the gene set required for the functional polarization of peritoneal macrophages [58]. Tissue-resident macrophages are programmed by local signals to express specific transcription factors that shape their identity. Other than having tissue-specific functions, resident macrophages monitor the tissue microenvironment, acting as sentinels for infections and tissue damage [58]. For example, *Lrg1*-expressing peritoneal macrophages are instructed in healthy tissues to continuously engulf apoptotic cells in an immunologically silent fashion [59]. Although the role of LRG1 in macrophages requires further investigation, these preliminary findings corroborate the hypothesis that LRG1 is part of the organism’s first line of defense and suggest its involvement in the maintenance of tissue homeostasis.

### LRG1 in wound healing and fibrosis

A growing body of evidence suggests that LRG1 is involved in physiological wound healing, although the full spectrum of its activities remains to be characterized. Wound healing is a complex and tightly regulated regenerative process which aims at restoring tissue integrity following infections, autoimmune diseases, as well as mechanical injuries. Healthy wound healing proceeds through overlapping phases, namely coagulation, inflammation, cell proliferation, ECM deposition and tissue remodeling, ultimately guiding the restoration of a functional epithelial barrier [60]. Moniruzzaman *et al.* reported that expression of the *Lrg1* gene, amongst others, mediates the proliferation of human colon epithelial cells in vitro following stimulation with IL-22 [61]. As IL-22 is a pro-inflammatory cytokine abundant in patients with ulcerative colitis (UC) that feature an extensively compromised intestinal mucosa, the hypothesis that LRG1 might orchestrate the correct renewal of damaged epithelial cells [62] is certainly very intriguing and in line with other observations. In fact, Pickert *et al*. previously demonstrated that STAT3 signalling, which in the intestinal epithelium is dependent on IL-22 rather than IL-6, is a key regulator of mucosal wound healing during acute experimental colitis and is associated with *Lrg1* upregulation [28]. Direct evidence that LRG1 plays a role in wound closure has been more recently provided by two independent studies showing that LRG1 accelerates keratinocyte migration over the wound bed by promoting stability of the re-epithelialization factor HIF-1α [63] and partial activation of the epithelial-to-mesenchymal transition (EMT) [56]. Interestingly, while exerting a direct migratory effect on wounded epithelial cells, LRG1 has also been reported to favour the healing of corneal epithelium through modulation of tissue-specific matrix metalloproteinases [64]. In addition, LRG1 enhances dermal angiogenesis and neutrophil extravasation, thereby contributing to the initiation of a beneficial inflammatory response at the site of injury [56]. While several cell types might contribute to LRG1 production, bone marrow-derived myeloid cells have been reported to represent a key source during both skin repair [56] and post-infarct myocardium remodeling [11].

Other than promoting wound healing, LRG1 may also contribute to the preservation of physiological tissue integrity. In fact, reduced levels of LRG1 following selective deletion of PPARβ/δ in fibroblasts, made the epidermis of mutant mice thicker and more susceptible to inflammation and dermal fibrosis [19]. Under normal conditions, structural abnormalities were not observed in other organs suggesting that the autocrine and paracrine effects of fibroblast-secreted LRG1 are likely to be localized and tissue specific. LRG1 has been shown to prevent the activation of skin fibroblasts by inhibiting pro-fibrotic TGFβ signalling [19]. A similar protective role has been described in the heart, where LRG1 is constitutively expressed via PPARβ/δ in resident fibroblasts to counteract TGFβ function and preserve tissue integrity [18]. Using tissue-engineered skin constructs, modelled in vitro with fibroblasts and keratinocytes, Rioux and colleagues examined the gene expression profiles from healthy and psoriatic skin biopsies. Interestingly, although *Lrg1* was identified as one of the genes most deregulated, its function was not clearly associated with any of the biological processes used for gene ontology assignment, including “keratinization” and “metabolic processes”, suggesting that the activities LRG1 exerts to restore or preserve skin integrity are still largely unknown [65].

Effective wound healing is based on a finely regulated crosstalk among different cell types. However, severe tissue damage, or the persistence of inflammatory stimuli, can cause aberrant cell signalling and either the formation of ulcerative defects (chronic wounds) or excessive ECM production (fibrosis) [60]. Abnormal levels of LRG1 in the skin of diabetic mice delay the closure of chronic wounds through the formation of NETs [56], whose dysregulated function is known to cause cell damage in a number of conditions including diabetes [66]. Similarly, LRG1 has been reported to promote fibrosis in lung [16, 67], kidney [68], dermal [34, 67, 69], and ocular tissues [57, 70, 71]. In particular, aberrant LRG1 levels were shown to promote ECM deposition both directly, through transactivation of resident fibroblasts [16, 68, 70] and epithelial cells [57, 71], and indirectly by disrupting the formation of functional vessels [34] and modulating neutrophil pro-fibrotic effects [57] (Fig. 5). Indeed, depletion of *Lrg1* was observed to protect against lung and skin fibrosis [16, 34, 67]. However, it is worth mentioning that while promoting fibrosis in some pathological settings, in others LRG1 exerts an inhibitory function on ECM deposition, which can be either beneficial [72] or detrimental by contributing to tissue deterioration [40], in a highly tissue-specific fashion.

Taken together these studies suggest, at least in some conditions, that LRG1 plays a crucial role in promoting physiological wound healing and maintaining tissue homeostasis. Nevertheless, its expression must be finely regulated as abnormal LRG1 levels appear to disturb effective wound healing and contribute to fibrosis and scarring possibly by altering the timely resolution of the inflammatory process.

### LRG1 in disease

In conditions of altered homeostasis that typically accompany disease, transcription of the *Lrg1* gene has been reported to be highly upregulated in endothelial cells [4, 70, 73–75], several types of epithelial cells [15, 17, 26, 28, 68, 76], fibroblasts [34, 57, 70, 77] and myeloid cells [11, 56, 72, 78–81] including microglia [82] (Fig. 5). Whilst our understanding of the full spectrum of LRG1 activities in pathology is still in its infancy, a considerable body of evidence is emerging to support the assertion that LRG1 mediates pathogenic mechanisms through acting on different cellular targets including endothelial, immune, epithelial, and mesenchymal cells (Fig. 5). However, the relative contribution made to various pathological processes by LRG1 derived from the liver *versus* that which is locally secreted remains to be fully determined.

**Fig. 6:**
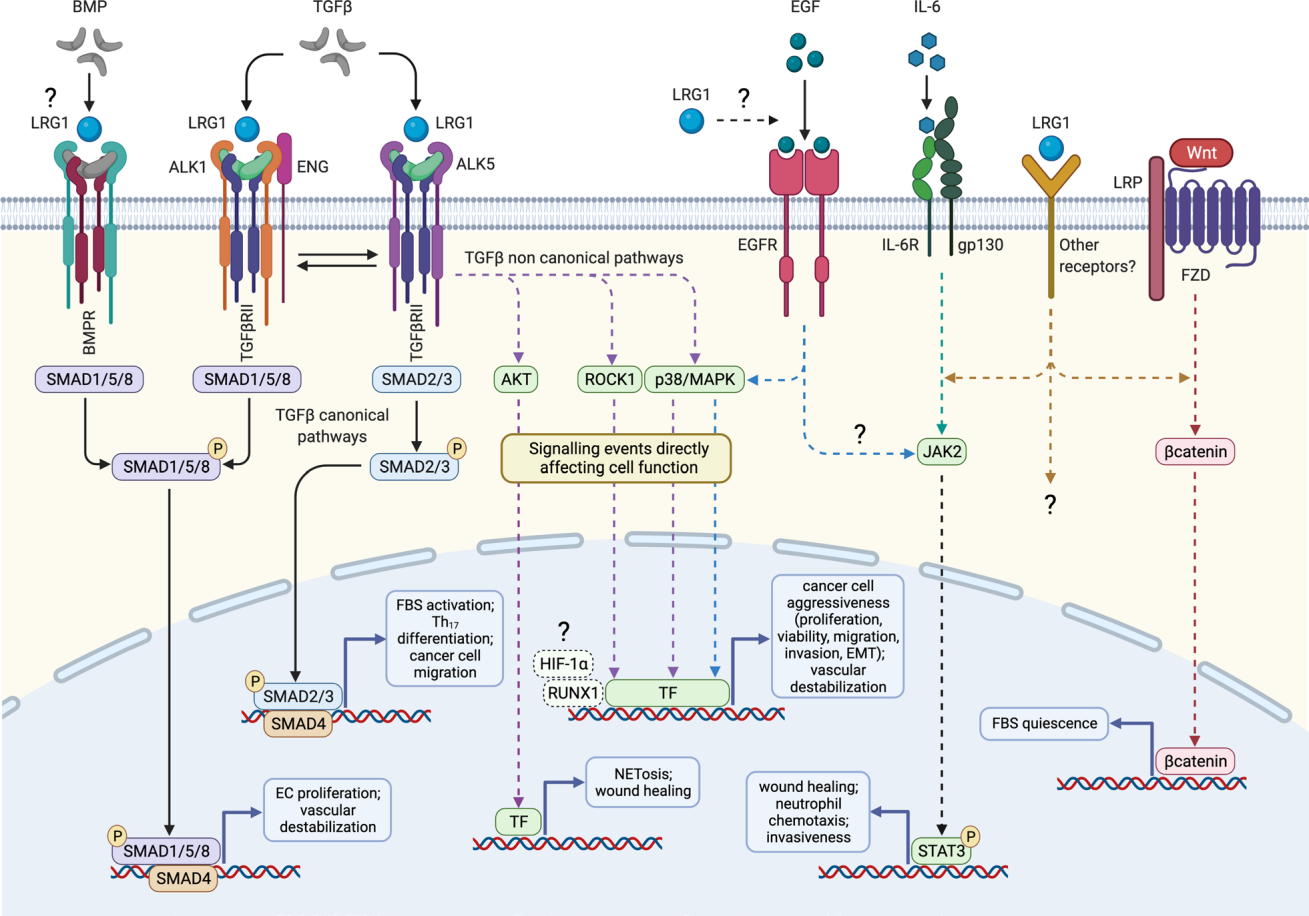
LRG1 signalling pathways. Simplified schematic representation of generic signalling pathways known, or speculated, to be modulated by LRG1 in a cell-specific manner. LRG1 likely modifies cell behaviour both directly, by altering the cell transcriptome, and indirectly by interfering with intermediate steps of the signalling cascades. LRG1 has been mainly described as a modifier of the TGFβ canonical pathway. While promoting pathogenic angiogenesis in endothelial cells through the ALK1-Smad1/5/8 pathway [4, 23], LRG1 may also modulate the ALK5-Smad2/3 arm to favour the formation of myofibroblasts [16, 68] and Th_17_ lymphocytes [27], thus sustaining fibrosis and inflammation, as well as glioma cell migration [194]. TGFβ non-canonical signalling is also likely to mediate some of the LRG1-driven biological functions including neutrophil activation and wound healing via AKT [56, 64], as well as the modulation of stem/cancer cell phenotype via ROCK1 [39] and p38/MAPK [31, 87]. Activation of the TGFβ-related transcription factors HIF-1α [197] and RUNX1 [50] has also been associated with LRG1 pro-oncogenic functions, although the specific upstream pathways subject to LRG1 modification in this context remain to be formally clarified. Additional transduction factors involved in LRG1 signalling include (i) EGFR which promotes pancreatic cancer cell malignancy through p38/MAPK [49], dissemination of melanoma cells [90] and cornea repair through STAT3 [64]; (ii) the IL-6/STAT3 axis which modulates neutrophil chemotaxis [57]; (iii) Wnt/βcatenin which, in the heart, inhibit fibroblast proliferation and migration [37]. Further investigations are needed to address whether LRG1 modulates the activity of other receptor-mediated signalling pathways including BMPs, and whether other receptors may also be directly or indirectly affected by LRG1. *BMP* bone morphogenic protein, *EC* endothelial cell, *FBS* fibroblast, *EMT* epithelial-mesenchymal transition, *FZD* Frizzled, *TF* transcription factor, *NET* neutrophil extracellular trap

One of the most compelling roles for LRG1 is its involvement in promoting diseased vessels in a wide variety of pathological settings, including diabetic nephropathy, diabetic retinopathy, age-related macular degeneration, and cancer. This role was first reported in 2013 when LRG1 was shown to be a novel regulator of pathogenic neovascularization through switching endothelial cell TGFβ signalling towards a proliferative pathway [4], commonly described as the TGFβ angiogenic switch. Simplistically, binding of TGFβ to endothelial TGFβ type II receptor (TGFβRII) can initiate canonical signalling through two different tyrosine kinase receptors, namely activin receptor-like kinase 1 (ALK1) and 5 (ALK5) [83]. Under physiological conditions, TGFβ activates in endothelial cells ALK5 and the transcription factors Smad2/3, which ultimately preserve cell quiescence [84]. However, high levels of LRG1 in the diseased *milieu* can redirect TGFβ to form a transduction complex with ALK1 and the accessory receptor endoglin (ENG), thus activating the pro-angiogenic Smad1/5/8 pathway and promoting endothelial cell proliferation, migration and tubulogenesis [4] (Fig. 6). Notably, ENG has been described as essential to augment the interaction between LRG1, TGFβ and ALK1 in endothelial cells while inhibiting the angiostatic arm of the TGFβ signalling [4, 85]. Moreover, in view of the role that LRG1 plays in delaying the onset of apoptosis by counteracting Cyt *c* cytotoxicity [42, 51], the hypothesis that LRG1 may exert a similar protective function in endothelial cells during angiogenesis is compelling and deserves further investigation. This linear view of TGFβ signalling does not, however, capture the true nature of the complex and often nuanced interactions that may influence outcomes in the most diverse settings. Indeed, in endothelial cells directing TGFβ signalling to a so-called pro-angiogenic pathway does not explain why such newly formed vessels grow in a disorganized and dysfunctional manner. This raises the possibility that LRG1 corrupts other key angiogenic processes which are responsible for vascular maturation and stability. For instance, data from our recent work on tumour vasculature [86] suggest that LRG1 may also disrupt the normal crosstalk between endothelial cells and pericytes, which is an essential prerequisite of vascular homeostasis, thus making vessels unstable and more prone to sprouting.

### LRG1-mediated pathological signalling pathways

The effects of LRG1 on TGFβ canonical signalling differ according to the pathological context and cellular target. While in endothelial cells LRG1 interacts with ALK1 and ENG to activate the pro-angiogenic Smad1/5/8 pathway [4, 23], in fibroblasts it promotes their differentiation into matrix-producing mesenchymal cells, and in lymphocytes the formation of pro-inflammatory Th_17_ cells, through phosphorylation of ALK5 and Smad2/3 in an ENG-independent manner [16, 27, 68] (Fig. 6). LRG1 has been reported to interfere with the TGFβ non-canonical pathway [31, 39, 56, 87] and, in all likelihood, it disturbs other members of the TGFβ superfamily as well, namely bone morphogenic proteins (BMPs), which also signal through Smad and non-Smad transduction cascades and whose impaired signalling similarly results in vascular defects [88] (Fig. 6). Additionally, LRG1 has been associated with the Wnt/βcatenin axis [37], for a long time implicated in pathological angiogenesis [89], as well as with the activation of the transcription factor STAT3 [48, 57, 64], which is a downstream effector of several signalling molecules including EGF, IL-6 and PDGF (Fig. 6). Indeed, epidermal growth factor receptor (EGFR) has been shown to mediate LRG1 activity in pancreatic cancer cells [49], metastatic melanoma cells [90], and corneal epithelium during wound repair [64], whereas the IL-6/STAT3 axis appears to modulate LRG1-driven neutrophil chemotaxis [57]. Therefore, one may speculate that LRG1 interferes with the activity of different receptors in a context-dependent manner and that functional outcomes will be most likely determined by the balance between various interacting pathways. At this juncture, it is worth noting that, while reporting an effect on the expression of specific signalling components, the majority of these studies do not clarify whether LRG1 acts also as a direct modifier of these cascades and, if so, whether it directly interacts with their upstream receptors or rather modulates the binding of other ligands. In fact, the observation that in naïve CD4^pos^ cells LRG1 promotes the expression of IL-6R through the TGFβ/Smad2 pathway points, at least in some contexts, to an indirect modulation of the signalling network [27]. Indeed, besides Cyt *c* [45], EGFR [49] and various members of the TGFβ transduction complex [4, 8, 91], no other signalling components have been formally recognized to date as ligands for LRG1. These data suggest that LRG1 exerts its pathogenic functions by modulating multiple transduction cascades and likely integrating them in a signalling network much more complex than originally postulated.

### Eye disease

A role for LRG1 in pathogenic ocular neovascularization was first reported in 2013 [4], when a significant increase in *Lrg1* expression was observed in laser-induced ocular choroidal neovascularization (CNV) in mice, and during the ischaemic proliferative phase of oxygen-induced retinopathy (OIR). These experimental models replicate aspects of neovascular age-related macular degeneration (nvAMD) and proliferative diabetic retinopathy (PDR), which is a major microvascular complication of diabetes mellitus. The contribution of LRG1 to these aberrant neovascular responses was illustrated by their partial inhibition following either genetic ablation of *Lrg1* or treatment with a LRG1 function-blocking antibody [4], an observation consistent with data confirming raised levels of LRG1 in human ocular disease [70, 92–95]. For instance, patients with PDR have significantly higher LRG1 levels in the vitreous than non-diabetic controls or diabetic patients without retinopathy [92, 93]. Increased LRG1 levels were also detected in the plasma of type 2 diabetes mellitus (T2DM) patients with PDR compared to patients without diabetes [92, 94], patients with T2DM without retinopathy and patients with non-proliferative diabetic retinopathy [92, 95]. It is not clear whether the increased concentration of LRG1 observed in the vitreous is caused by local production or leakage from the systemic circulation. However, the fact that experimentally *Lrg1* expression is induced in diseased ocular tissues [4] points, at least partly, to local production. Similarly, and consistent with a model of LRG1 production at the site of lesion and subsequent release into the fluidic ocular compartments, higher levels of LRG1 have been recently observed in choroidal neovascular membranes and aqueous samples of treatment-naïve nvAMD patients [70]. This study also revealed that whereas subretinal neovessels of all naïve nvAMD patients express high levels of LRG1, an inconsistent production is observed in those who receive anti-VEGF treatment. Thus, it is intriguing to speculate that LRG1 expressed from endothelial cells might be switched off through anti-VEGF mediated endothelial cell normalization resulting in an excellent response to treatment. However, patients where LRG1 continues to be expressed, possibly through persistent production from myofibroblasts or even microglia, may represent those who are known to respond poorly to anti-VEGF treatment.

In addition to the TGFβ-mediated pro-angiogenic effect previously discussed, a connection between LRG1 and the renin-angiotensin system (RAS) in PDR has been suggested. RAS plays an important role in pathological angiogenesis and microvascular diabetic complications [96]. LRG1 plasma levels have been shown to correlate with the soluble form of the (pro)renin receptor, which is a well-known RAS initiator. It is still unclear, though, whether there is a direct interaction between RAS and LRG1 as the latter does not appear to promote the expression of the soluble (pro)renin receptor in human retinal microvascular endothelial cells [94, 97].

The pathogenic activity of LRG1 has been further confirmed in the corneal alkali burn mouse model, where LRG1 facilitates corneal angiogenesis and lymphangiogenesis through enhancing the stromal production of all VEGFs and VEGFR isoforms [98]. In line with this, a reduction of *Vegfa* expression in the retina of 3 months old *Lrg1* knockout mice had already been reported [4], although with marginal and reversible effects on vessel development. Increased LRG1 levels were observed both in the epithelium and the stromal compartment of alkali-burned corneas, and associated with a more severe fibrotic response [57].

These data have led to the proposed therapeutic targeting of LRG1 in patients with nvAMD, diabetic retinopathy and diabetic macular oedema on its own or as an adjunct therapy [99].

### Kidney disease

A severe microvascular complication of diabetes mellitus is diabetic kidney disease (DKD). Early stages of DKD, also referred to as diabetic nephropathy, are characterized by proliferation of immature and leaky vessels, whereas later stages are dominated by capillary rarefaction and fibrosis [100]. In mice and humans with DKD, LRG1 expression is increased in glomerular endothelial cells [23, 24, 101] prior to the upregulation of VEGF [24], which corroborates the view of LRG1 being an early pathogenic factor driving initial microvascular instability and priming the vasculature for angiogenesis. Indeed, *Lrg1* deletion was found to be protective against DKD as *Lrg1*^*−/−*^ streptozotocin (STZ)-induced diabetic mice show milder angiogenesis, reduced podocyte foot process effacement and significantly improved kidney function. Similar to observations in ocular neovascularization [4], LRG1 directs the TGFβ signalling towards the Smad1/5/8 pro-angiogenic pathway in glomerular endothelial cells under diabetic conditions [23]. Interestingly, although less prominent, an increase in LRG1 expression was also observed in the tubulointerstitial compartment of diabetic kidneys. Since a recent study demonstrated that LRG1 secreted by epithelial cells exacerbates fibrosis in a mouse model of kidney obstruction by amplifying the TGFβ/Smad3 signalling in resident fibroblasts [68], it is reasonable to speculate that a similar mechanism may occur in the fibrotic stage of diabetic and, in general, chronic kidney disease overall. The observation that higher plasma LRG1 levels were associated with faster decline of kidney function in T2DM patients was also reported in other studies [102, 103] lending further weight to the hypothesis that LRG1 inhibition may deliver therapeutic benefits.

Besides DKD, other renal conditions have been associated with abnormal LRG1 secretion and include IgA nephropathy, where increased urinary LRG1 levels have been shown to correlate with disease severity, and paediatric idiopathic nephrotic syndrome [104, 105]. Moreover, higher levels of LRG1 have been detected in urine of mice following renal injury and increased production by renal tubular epithelial cells [106].

### Lung disease

LRG1 has been suggested as a reliable biomarker for the diagnosis and monitoring of dermatomyositis-associated pneumonia [107] and airway inflammation in asthma [15]. In a mouse model of ovalbumin-induced asthma, LRG1 was shown by immunohistochemistry to be secreted locally by putative non-ciliated, mucin-producing bronchial epithelial cells, known as goblet cells, which under physiological conditions are not known to express LRG1. Moreover, the authors demonstrated in vitro that LRG1 expression by normal bronchial epithelial cells requires IL13-driven transdifferentiation into goblet cells, a process referred to as goblet cell metaplasia and responsible for the excessive mucous production observed in asthmatic patients [15]. As previously shown in the inflamed colon [28, 76], this study supports the hypothesis that mucosal epithelial cells represent a source of LRG1 during inflammatory reactions. Locally secreted LRG1 might play a direct role in the pathogenesis of asthma by triggering TGFβ-induced subepithelial fibrosis [15]. Indeed, in a mouse model of idiopathic pulmonary fibrosis, LRG1 was shown to activate ECM-producing lung fibroblasts through the TGFβ/Smad2 pathway in an ENG-independent manner [16] (Fig. 5).

Bronchopulmonary dysplasia (BPD) is a condition affecting the lungs of premature infants caused by a massive mechanical ventilation-induced inflammatory response, which ultimately impairs vascular development [108]. RNA-seq studies showed significantly higher expression of *Lrg1* in pulmonary endothelial cells isolated from LPS-treated wild-type newborns [33], which recapitulate the clinical signs of BPD. This observation suggests that, although LRG1 seems to be dispensable for normal development, inflammation-induced high levels of LRG1 might be responsible for aberrant vasculogenesis even in the developing lung. Recently, in a seminal study by Hisata and colleagues [74], the contribution of LRG1 to pulmonary microvascular dysfunction has been confirmed in chronic obstructive pulmonary disease (COPD)/emphysema, a condition characterized by gradual loss of endothelial cells and capillary rarefaction ultimately leading to respiratory failure. In this milestone study, the authors showed that LRG1 levels are upregulated in human COPD samples and positively correlate with the severity of COPD phenotype. They further demonstrated that conditional deletion of *Lrg1* in endothelial cells is sufficient to rescue the phenotype of lung vessels and organ functionality in a mouse model of emphysema, formally proving that LRG1 drives tissue malfunction through destabilization of the endothelial compartment [74].

### Cardiovascular disease

Whilst raised levels of LRG1 have been mostly associated with disease progression, its contribution to cardiovascular disease is less clear with both protective and pathogenic properties being reported.

In response to injury caused by various cardiovascular events, for example tissue infarction, the myocardium undergoes several pathological changes that include inflammation, abnormal ECM deposition and loss of terminal capillaries, which eventually lead to heart failure [109]. In a mouse model of myocardial infarction, cardiac LRG1 levels significantly increased during the acute inflammatory response before gradually declining as tissue remodelling progressed towards the fibroproliferative phase [11, 37]. In contrast, *Lrg1* deletion was associated with impaired perfusion, increased ECM deposition and reduced organ functionality [11]. Of note, local upregulation of LRG1 was also observed following peripheral ischemic events [79]. A recent study suggests that LRG1, produced at least partly by the endothelium of stenotic arteries in response to TNFα and atherogenic flow, might serve as a negative regulator of the inflammatory process by modulating the shedding of TNF receptor 1 and inhibiting the expression of the monocyte recruitment proteins VCAM-1 and ICAM-1 [73]. Moreover, LRG1 has been shown to induce autophagy and apoptosis in rat cardiomyocytes under hypoxic conditions, through the expression of HIF-1α [110]. During the initial phase of cardiac ischemia, characterized by progressive oxygen deprivation, autophagy has been described as a key compensatory mechanism aimed at maintaining the energy balance through alternative ATP generation [111]. Similarly, during the reperfusion phase when dysfunctional mitochondria may cause reactive oxygen species overproduction and further tissue damage, mitochondrial autophagy, referred to as *mitophagy*, can be induced as a protective response [111]. Consistent with the hypothesis of LRG1 exerting some beneficial functions in tissue healing, it is reasonable to speculate that LRG1 might be part of these adaptive metabolic responses which ultimately support cardiomyocyte survival after infarction. However, it must be noted that excessive autophagy can induce cell death not only through degradation of necessary cell components but also by interfering with the expression of the survival factor Bcl-2 [111]. This could explain the apparently contradictory effects LRG1 plays by promoting both autophagy and apoptosis in cardiomyocytes and suggests that, like all the other *bona fide* pro-inflammatory molecules, its function is highly dose-dependent and thus requires tight regulation. Decreased LRG1 levels have also been detected in the heart of aged mice and several conditions characterized by excessive cardiac fibrosis, which is known to increase tissue stiffness and impair muscle performance [112]. Indeed, similarly to what is observed in the post-infarct myocardium, LRG1 has been shown to counteract ECM deposition and improve organ functionality in a mouse model of aortic constriction-induced cardiac fibrosis [18]. Taken together these observations suggest that, at least in the heart, local upregulation of LRG1 might be part of a compensatory response which, following tissue damage, favours myocyte survival, promotes beneficial revascularization and counteracts abnormal tissue remodelling, either indirectly through improved perfusion or directly by neutralizing the pro-fibrotic TGFβ pathways [18].

While exerting putative protective functions locally, serum LRG1 has been suggested as a valuable biomarker for the diagnosis and monitoring of various cardiovascular diseases. However, whether systemic LRG1 upregulation primes the heart and somehow influences its response to other pathogenic mechanisms remains to be formally clarified. In a large study, including over 2000 patients with T2DM, higher plasma levels of LRG1 were significantly associated with peripheral artery disease and several cardiovascular risk factors including arterial stiffness, endothelial dysfunction, systolic blood pressure, age, obesity, kidney function and high-sensitivity C-reactive protein (hsCRP). Notably, female study participants showed higher LRG1 levels than males [113, 114] which supports other findings suggesting there are sex differences in the biology of LRG1. Other studies reported a positive correlation between circulating or coronary sinus LRG1 levels and the incidence of heart failure [115–118] and idiopathic pulmonary arterial hypertension [119]. In contrast, in patients with statin-treated familial hypercholesteremia, detectable serum levels of LRG1 were observed only in the absence of coronary artery disease [120]. The authors speculate that LRG1 levels might be systemically increased only in the early stage of cardiovascular disease, which is characterized by arterial stiffening and endothelial dysfunction, while declining during disease progression. In support of this hypothesis, LRG1 circulating levels were shown to be predictive of early atherosclerotic events but not of late-stage myocardial infarction [121].

Endothelial dysfunction is a characteristic of early atherosclerosis [113] and predicts future cardiovascular events [122]. The ENG receptor is known to promote vasodilation by increasing nitric oxide production in endothelial cells through the Smad2 pathway [123]. Since LRG1 has been widely reported to direct endothelial TGFβ signalling towards the Smad1/5/8 pathway through ENG, it has been postulated that LRG1 might hinder normal endothelium-dependent vasodilation by inhibiting the Smad2 pathway and ultimately reducing the bioavailability of nitric oxide [113]. Endothelial dysfunction is, among other factors, caused by chronic systemic low-grade inflammation, which is recognized by itself as a major risk factor for the development of cardiovascular disease [124]. Considering the compelling role for LRG1 in various inflammatory conditions, as later discussed, it is tempting to speculate that LRG1 circulating levels might be upregulated also in the context of chronic systemic inflammation, thus contributing to systemic blood vessel dysregulation. In chronic kidney disease, for example, inflammation promotes atherosclerosis and is associated with a high incidence of cardiovascular disease. In patients with end-stage renal disease, LRG1 significantly correlates with other markers of inflammation such as IL-6 and hsCRP, as well as T cell immunosenescence, and increased LRG1 levels are independently associated with the presence of peripheral arterial occlusive disease and cardiovascular disease [125].

### Diabetes

As detailed in previous sections, studies on diabetic patients and animal models have provided us with compelling evidence linking LRG1 to vascular dysfunction. It is well accepted that diabetes has injurious effects on the vasculature resulting in microvascular (retinopathy, nephropathy, and neuropathy) and macrovascular (coronary artery disease, peripheral arterial disease and stroke) complications which represent major causes of morbidity and mortality in diabetic patients [126].

To date, no convincing evidence exists to show whether circulating LRG1 levels are increased in diabetic patients prior to any micro- or macrovascular clinical disease. Indeed, in normal control and T2DM patients without diabetic retinopathy no significant differences in plasma LRG1 levels have been observed suggesting no early contribution [92]. In another study, increased urinary LRG1 levels in type 1 diabetes mellitus (T1DM) patients were found compared to healthy siblings. Of note, although none of these patients had any sign of kidney function impairment, the authors also observed higher levels of urinary lysosomal proteins, which have been suggested as early markers for subclinical kidney disease [127]. It is therefore unclear whether the increased urinary LRG1 levels stem from systemic circulation or local production in the kidney and whether they may be indicative of early pre-clinical disease.

Increased LRG1 levels have been found in the plasma of T2DM patients and described as statistically significant predictors of peripheral arterial disease [113]. In the kidney, dysfunction is also a major co-morbidity of long-term diabetes, so it is particularly relevant that in a 3-year prospective study, higher circulating levels of LRG1 were predictive of diabetic nephropathy progression in T2DM patients [102]. Interestingly, a recent study revealed a substantial increase of LRG1 also in urine samples of young T1DM patients. This finding provided the first evidence that LRG1 expression could be switched on early in the disease and that it is not exclusively linked to high body mass index (BMI) or obesity, features more often associated with T2DM patients [127]. Overall, compelling evidence now exists that LRG1 predicts all-cause and cause-specific mortality risk in diabetic patients [128].

To date, most studies on circulating levels of LRG1 in diabetic patients are cross-sectional, leaving it unclear whether elevated levels of this molecule represent cause or consequence of diabetic vascular complications. Animal studies would certainly suggest the former, with *Lrg1*^−/−^ mice exhibiting a milder phenotype in models of PDR [4], diabetic kidney disease [23, 24, 101] and diabetic wound healing [56]. Despite the limitations intrinsic to mimicking a complex chronic endocrine disorder in rodents, these studies have revealed various mechanisms through which LRG1 could be pathogenic in the context of diabetic vascular complications. For instance, LRG1 promotion of pathological angiogenesis has been described in animal models of diabetic retinopathy and kidney disease with retinal and glomerular endothelial cells being the source of LRG1, respectively [4, 23, 24, 101]. Additionally, LRG1 has been shown to regulate NETosis, a process known to characterize and delay diabetic wound healing [56]. However, LRG1 expression was downregulated in corneal keratinocytes of diabetic mice [64] while topical application of recombinant LRG1 was shown to accelerate the re-epithelization of corneal wounds [64], again illustrating the context-dependent nature of LRG1 effects. The fact that the cornea is an avascular tissue, while the retina and kidney are strongly vascularized, suggests that elevated LRG1 expression could be triggered by circulating factors such as advanced-glycation end (AGE) products, abundant in the plasma of diabetic patients [129]. Moreover, circulating pro-inflammatory cytokines such as IL-6, markedly present in T1 and T2DM patients, could trigger both local and systemic increased LRG1 expression [130].

The current therapeutic strategy for diabetic patients is based on the reduction of blood glucose levels through pharmacological and/or lifestyle/dietary interventions, concomitantly with the pharmacological lowering of hypertension in patients at high risk of cardiovascular events. Regrettably, glycemic control alone is unable to completely halt or revert the occurrence of micro- and macro-vascular complications, which remain the main cause of morbidity and mortality in these individuals. In the case of diabetic-induced ocular vascular problems, agents blocking VEGF signalling have revolutionized the treatment of PDR and diabetic macular oedema but do not show efficacy in all patients and their effects may be short-lived, suggestive of alternative pathways at play [131, 132]. Similarly, in diabetic nephropathy, AGE-inhibitors, used as first-line therapeutic agents, are only effective in approximately half of patients [133]. These observations would indicate contributing pathogenic pathways and, given the pre-clinical data currently available, it is reasonable to speculate that LRG1 may drive, at least partly, disease progression in standard-of-care treated refractive patients.

Obesity, which is often characterized by chronic low-grade inflammation [134], plays a critical role in the pathogenesis of various metabolic disorders including insulin resistance, a major risk factor for T2DM. LRG1 levels are significantly higher in the serum and fat depots of obese humans [22] and positively correlate with BMI, waist circumference and visceral fat mass [113]. He and colleagues recently uncovered a novel pathogenic role for LRG1 by demonstrating its crucial contribution to high fat diet (HFD) metabolic dysfunctions [22]. The authors showed that high levels of circulating LRG1, produced at least partly by distant organ adipocytes, can exacerbate the hepatosteatosis and insulin resistance often observed in HFD by corrupting the normal function of hepatocytes. In vitro observations suggest that LRG1 may promote: i) lipid accumulation by suppressing fatty acid catabolism and inducing lipid biosynthesis through activation of the transcription factor SREBP1, and ii) hyperglycemia by downregulating the expression of the insulin receptor substrates IRS1 and IRS2 [22] (Fig. 5). Interestingly, although further investigations are required to dissect the mechanisms underlying these pathogenic outcomes, the data reported suggest that the inhibitory effect exerted by LRG1 on hepatic insulin signalling is independent of TGFβ [22].

Work carried out in *db/db* mice, which are insulin-resistant and commonly used as a mouse model for T2DM, reveals that *Lrg1* expression is downregulated in white adipose tissue following treatment with PPARγ agonists [135], anti-inflammatory drugs known to increase insulin sensitivity [136]. This, together with the observation that circulating LRG1 preferably binds to hepatocytes [22], supports the hypothesis of LRG1 being a novel adipokine orchestrating an almost exclusive metabolic crosstalk between adipose tissue and liver in obesity. Whether LRG1 exerts metabolic functions also in physiology remains unclear. However, the fact that *Lrg1* knockout mice show reduced body weight gain and smaller adipocyte cell size in HFD feeding conditions [22], and that the *Lrg1* gene is switched on during development concurrently with adipocyte lipogenesis, points towards a role in the regulation of energy homeostasis.

### Inflammatory disorders

In the past decade the development of advanced proteomic techniques [137] made it progressively more evident that LRG1 expression is enhanced in a plethora of inflammatory disorders. Among those, autoimmune diseases are particularly disabling conditions representing a major hurdle both in terms of diagnosis and treatment. In particular, serum LRG1 has been identified as a useful biomarker for monitoring disease activity in patients with adult-onset Still's disease [138], psoriasis [65, 139, 140] lupus nephritis [17], rheumatoid arthritis [141–144] and vasculitis [145–149]. However, the role of LRG1 in these conditions remains largely unclear. Nevertheless, the identification of specific serum biomarkers able to mirror disease progression is key and avoids the reliance on invasive tissue biopsies, such as in the case of kidney disease. A recent study demonstrated that high levels of LRG1 in the plasma of patients affected by lupus nephritis correlate with poor renal function [17]. As hyperplasia of renal endothelial cells represents one of the most crucial pathological changes occurring in lupus nephritis, it is tempting to speculate that LRG1-driven aberrant angiogenesis might represent a contributing factor, although further studies are needed to confirm this hypothesis. The role of LRG1 as a pro-inflammatory mediator is more clearly established in the pathogenesis of rheumatoid arthritis (RA), where high levels of Th_17_ cells and Th_17_-related cytokines, including TNFα and IL-6, cause severe joint destruction and correlate with poor prognosis [150–152]. It is known that following stimulation with TGFβ, naïve CD4^pos^ T cells differentiate into anti-inflammatory T_reg_ lymphocytes [153]. However, when high levels of IL-6 are present in the inflammatory *milieu*, TGFβ rather sustains the formation of pro-inflammatory Th_17_ cells [154]. Urushima *et al*. demonstrated that LRG1 can modulate the TGFβ pathway in naïve CD4^pos^ T cells by enhancing their polarization into both phenotypes depending on the surrounding cytokines [27]. LRG1 was reported to promote, through the TGFβ/Smad2 axis, the expression of IL-6R on naïve CD4^pos^ T cells, therefore boosting the IL-6/STAT3 pathway and the formation of pro-inflammatory Th_17_ cells (Fig. 5). Indeed, in a collagen-induced experimental model of arthritis, *Lrg1* ablation reduced severity of symptoms and protected mice from cartilage destruction by inhibition of Th_17_ differentiation [27].

Abnormal LRG1 levels have also been detected in several inflammatory disorders associated with the gastrointestinal tract [155]. For instance, a substantial increase in *Lrg1* expression was observed in mouse models with inflammation of the cystic fibrosis intestine [156] and severe colitis [157, 158]. Using a chemically induced model of chronic colitis mirroring all the stages of the disease, Wu and Chakravarti showed that *Lrg1* expression was highly upregulated during the inflammatory peak before rapidly declining to normal levels. Interestingly, several pro-fibrotic genes were also upregulated during this stage, but their overexpression persisted after the inflammation had subsided [158], suggesting that LRG1 is not physiologically involved in ECM remodeling. Also, in patients affected by UC, serum LRG1 has been described as a useful biomarker to evaluate disease activity, and histological analysis of surgically resected colons confirmed that LRG1 is expressed in epithelial cells within inflamed lesions [76].

Dysregulated angiogenesis is known to play a key role in the progression of osteoarthritis (OA), where neovessels in the subchondral bone invade the overlying articular cartilage and indirectly facilitate de novo bone formation through oxygen and nutrient supply. In a mouse model of OA, *Lrg1* expression was upregulated in both the subchondral bone and articular cartilage and was associated with higher numbers of CD31^pos^ cells and bone-committed mesenchymal progenitors [31]. While confirming the pro-angiogenic effects of LRG1 on endothelial cells, the authors also demonstrated in vitro that LRG1 can induce migration of bone marrow mesenchymal stem cells through the MAPK/p38 signalling pathway.

These studies substantiate results from other diseases where, in contrast to *bona fide* APPs which are produced exclusively by the liver, LRG1 is also synthesized at sites of injury. C-reactive protein (CRP) and serum amyloid A protein (SAA) are non-redundantly regulated by IL-6 [159] and therefore are not suitable to monitor the inflammatory status or concomitant infections in patients receiving IL-6 blocking drugs. Moreover, in some diseases like UC, IL-6 does not play a major role as CRP and SAA often remain at physiological levels [30]. Thus, the development of novel biomarkers represents an urgent clinical need for the early diagnosis and management of some inflammatory diseases and LRG1, being regulated by multiple pro-inflammatory cytokines, stands as a very promising candidate [160].

### Infections

In human, LRG1 serum levels have been reported to rise following bacterial infections with *Haemophilus influenzae type b*, *Salmonella*, *Streptococcus pyogenes* [161], *Staphylococcus aureus* [52] and *Mycobacterium tuberculosis* [162, 163]. Furthermore, increased LRG1 levels have been found in the blood of children with appendicitis [164] and in adults with sepsis [165].

Higher levels of circulating LRG1 have also been detected in patients with chicken pox, measles, mumps [161], severe acute respiratory syndrome (SARS) [166] and HIV infection [52]. In support of this observation, lower levels of LRG1 have been observed in a cohort of HIV elite suppressors, rare individuals who are able to control viremia by their natural immunological mechanisms without highly active antiretroviral therapy [167]. A recent study has reported that not only is LRG1 significantly upregulated in patients affected by the newly discovered SARS-CoV-2 virus, but that it can also help distinguish between mild and severe cases [168, 169]. Strikingly, the latter are characterized by having high circulating levels of IL-6, which is known to be a key activator of *Lrg1* expression. Indeed, lung microvascular damage has been recognized as one of the major contributing factors to the pathogenesis of COVID-19 [170]. In addition, it has been suggested that excessive neutrophil function and dysregulated NETosis significantly contribute to inflammation and microangiopathy [171]. Given the well-described involvement of LRG1 in promoting diseased vessels and its newly recognized function in pathogenic NETosis, it is not unreasonable to speculate that LRG1, produced in response to IL-6 and most likely other pro-inflammatory cytokines, might represent a viable prognostic and therapeutic target for the treatment of vascular problems associated with SARS-CoV-2 infection.

### Cancer

Of all the conditions in which LRG1 has been implicated, the clinical evidence for a role in cancer has become the most overwhelmingly compelling (Fig. 3B, F). With the exception of few publications reporting a putative protective role in tumour progression [172–175], a consistent number of proteomic studies have identified LRG1 as a valuable biomarker for the diagnosis and clinical assessment of a variety of cancer types (Table 1). LRG1 circulating levels were shown to correlate, alone or in combination with other markers, with disease progression, burden, and poor prognosis [176–184]. Moreover, LRG1 has been described as a powerful complementary marker to differentiate early-stage tumours from benign lesions and healthy controls [182, 185–189]. Immunohistochemical detection of LRG1 in tissue sections has also shown that local tumour expression correlates with disease progression and patient survival [49, 180, 181, 183, 190]. Following profiling of the tumour epithelial glycoproteome, Surinova *et al*. validated LRG1 as a tumour-derived blood biomarker and its utility as a potential diagnostic tool for colorectal cancer (CRC) [191], confirming that tumours may themselves be a source of circulating LRG1 [183, 186]. Moreover, as larger tumours secrete more proteins into the circulation, LRG1 blood levels have been shown to positively correlate with tumour size [191, 192] further paving the way for LRG1 to stand as an accurate and tumour-related circulating biomarker with high diagnostic and prognostic value. Notably, data collected during a retrospective study revealed higher circulating levels of LRG1 in the plasma of subjects who were subsequently diagnosed with CRC, therefore suggesting that LRG1 might be also predictive of cancer onset [193].

**Table 1:** LRG1 expression in human cancer

	Sample	Role of LRG1	References
Biliary tract	Serum	In conjunction with CA19-9 and IL-6, particularly elevated in high-risk patients with primary sclerosing cholangitis	[179]
	Tumour tissue	Suggested as independent prognostic factor	[224]
Bladder	Urine	Suggested as biomarker for early diagnosis and monitoring of recurrence	[186]
Breast	Tumour tissue	Suggested as biomarker for neo-adjuvant aromatase inhibitor treatment. Associated with number of lymphatic metastasis, tumour stage and poor survival	[225, 226]
Cervical	Urine	Together with MMRN1, highly expressed in urines of cervical cancer patients	[227]
Colorectal	Plasma	In conjunction with other biomarkers, proposed as predictive, diagnostic, and prognostic factor. Positively correlates with tumour stage and size	[184, 191, 193, 228, 229]
	Serum	In conjunction with other biomarkers, suggested as diagnostic, prognostic, and follow-up factor. Associated with altered glycosylation	[13, 230–233]
	Tumour tissue	Associated with cancer aggressiveness and vascular density. Proposed as diagnostic in general and prognostic for stage III colorectal cancer	[180, 184, 197]
	Stool	In conjunction with Hp, SYNE2, RBP4, FN1 and ANXA6, suggested for early detection of high-risk adenomas and colorectal cancer	[234]
Endometrial	Tumour tissue	Suggested as independent prognostic factor of stage and lymphatic metastasis	[235]
Esophageal	Plasma	Significantly elevated in esophageal squamous cell carcinoma and, in conjunction with alpha-2-HS-glycoprotein, proposed as biomarker for early diagnosis	[236]
	Tumour tissue	Closely correlated with worse clinical survival	[237]
	Serum	In combination with CRP and sIL-6R, suggested as biomarker to predict response to preoperative chemoradiotherapy	[238]
Gastric	Serum and tumour tissue	Proposed as prognostic factor. Promotes tumour progression and affects negatively patient relapse-free survival. Correlation between tissue scores and serum levels	[183]
Glioblastoma	Plasma	In conjunction with CRP and C9, shows positive correlation with tumour size	[192]
	Tumour tissue	Significantly higher than in lower-grade glioma. Proposed as potential diagnostic, prognostic, and regional biomarker	[239]
Hepatocellular	Serum	Significantly elevated as part of a broad panel of protein biomarkers and associated with poor responders followig transarterial chemoembolization	[240, 241]
	Tumour tissue	Positive correlation with tumour size, differentiation, stage, vascularity. Negative correlation with patient survival	[181]
Leukemia	Serum	Highly expressed in acute lymphoblastic leukemia T and B cells. Suggested as biomarker for early diagnosis	[189, 242]
Lung	Plasma	Significantly elevated in patients with non-small cell lung carcinoma and, in conjunction with ACT, C9 and Hpt, proposed as diagnostic factor. Highly indicative of reduced survival time post-radiotherapy	[178, 243]
	Serum	Significantly elevated and, in conjunction with SAA and C4BP, prognostic in patients with squamous cell lung carcinoma. Expressed by circulating tumour cells in metastatic patients	[244, 245]
	Urine	Candidate biomarker for diagnosis of non-small cell lung carcinoma. Highly expressed in urinary exosomes	[246, 247]
	Tumour tissue	Upregulated in non-small cell lung carcinoma	[190]
Oral	Plasma	In combination with apolipoprotein A-IV, suggested as biomarker for oral cancer screening and early diagnosis	[248]
	Serum	Increased in oral squamous cell carcinoma and suggested as early diagnostic tool with ABG, CLU, PRO2044, HAP, C3, proapo-A1 and RBP4	[249]
	Saliva	Significantly elevated in oral squamous cell carcinoma and, together with CFB, C3, C4B and SERPINA1, associated with increased risk	[250]
Ovarian	Serum	Alone or in combination with other biomarkers suggested as diagnostic factor	[176, 182, 187, 251]
	Urine	Multiple LRG1 peptides detected in the urines of all ovarian cancer patients	[227, 252]
Pancreatic	Plasma	Exceeds diagnostic performance of CA19-9 alone in the early detection of pancreatic ductal adenocarcinoma (PDAC). High levels distinguish PDAC from chronic pancreatitis. Elevated during formation of intraductal papillary mucinous neoplasm	[185, 253–258]
	Serum	In combination with CA19-9, suggested as diagnostic biomarker. Characterized with altered glycosylation pattern. Increases with clinical stage	[12, 177, 259]
	Tumour tissue	Associated with higher recurrence rate and worse recurrence-free survival	[256]
Prostate	Serum	Elevated in fatal prostate cancer. Positively correlated with high risk of disease progression and mortality	[260]
Renal	Tumour tissue	Overexpressed in clear renal cell carcinoma and negatively related to patient survival	[261]
Retinal	Tumour tissue	Highly expressed in retinoblastoma	[262]

List of publications where LRG1 is discussed as a potential biomarker for the diagnosis, prognosis and monitoring of various cancer types. The samples used for LRG1 detection (serum, plasma, tissue section or urine), together with the main findings reported in the studies, are included.

Tumour cells, however, are not the only source of LRG1. In the tumour microenvironment (TME) stromal cells, including endothelial and myeloid cells, also contribute to LRG1 production (Fig. 3B, F). Several in vitro studies suggest that LRG1 acts directly on tumour cells enhancing their viability, proliferation, and invasive properties [39, 48, 49, 77, 87, 183, 184, 194–196] (Fig. 5). In CRC, for example, LRG1 has been shown to inhibit apoptosis and modulate the EMT of tumour cells through expression of the transcription factors RUNX1 [50] and HIF-1α [197], whose association with TGFβ and contribution to tumour growth are well-established (Fig. 6). Indeed, as LRG1 has been described as a modifier of TGFβ signalling, this raises many questions over its potential role in tumour progression. In fact, TGFβ is predominantly suppressive in the early pre-malignant stages of tumour development but can switch to being pro-oncogenic enhancing EMT, tumour invasion, metastatic dissemination and evasion of the immune system [198]. This has been referred to as the TGFβ paradox but how it occurs is not fully understood. Nevertheless, it raises the possibility that LRG1 may favour tumour aggressiveness through promotion of various TGFβ pro-oncogenic functions [39, 199]. For example, in human hepatoma cells, induction of LRG1 expression has been associated with loss of TGFβ-mediated growth inhibition [199].

Aside from its direct effect on cancer cells, TGFβ is also implicated in metastatic tumour cell survival through its effect on stromal cells, including cancer-associated fibroblasts (CAFs). This is especially germane in CRC where mutational inactivation of TGFβ signalling pathways occurs alongside a paradoxical upregulation of TGFβ production. Evidence suggests that the increased TGFβ production observed in TGFβ-unresponsive CRC cells acts selectively on neighboring stromal cells rendering them more susceptible to CRC cell colonization [200]. As CRC cells express also high levels of LRG1, one could hypothesize that this may contribute to the pro-oncogenic activity typically acquired by stromal cells in response to high levels of TGFβ. Moreover, it has been recently reported that CAFs themselves represent a source of LRG1 and that this can directly affect the invasive properties of CRC cells [77], thus placing LRG1 at the center of a mutual crosstalk between stromal and cancer cells in the metastatic TME.

As LRG1 has been shown to modify TGFβ non-canonical signalling in other pathological settings, it is not surprising that in cancer it can also exert its bioactivities through the p38/MAPK cascade [39, 87]. Moreover, recent evidence suggests that LRG1 might target cancer cells not only through the TGFβ signalling pathway [194] but also by selective interaction with EGFR [49] (Fig. 6). The crucial contribution of LRG1 to tumour growth has been further confirmed in vivo where ablation [195] or overexpression [49] of *Lrg1* in cancer cells respectively delayed or promoted growth of xenograft tumours.

De novo angiogenesis is crucial to support tumour growth and metastasis to distant organs. Therefore, therapeutic targeting of the master regulator of angiogenesis, namely VEGF, became a major focus to restrict vessel growth and hence limit tumour expansion. Whilst such approaches have shown some success in increasing progression-free survival, they have been largely disappointing in improving overall survival rate. As widely discussed across this review, LRG1 orchestrates pathological blood vessel formation [29] by enhancing proliferation, migration and invasion of endothelial cells [35], as well as the expression of several pro-angiogenic factors including TGFβ, VEGFA and Angiopoietin-1, in both endothelial [35, 190] and cancer cells [197]. A recent study on tumour vessel co-option, where tumours grow around existing vessels rather than through neoangiogenesis, revealed that *Lrg1* was one of the few genes significantly upregulated in endothelial cells, as both endothelial and pericyte transcriptomes were otherwise largely indistinguishable from those of normal vessels [201]. The effects of LRG1 on the cancer vasculature are therefore most likely inevitable and supportive of tumour progression. Indeed, we observed in a number of mouse tumour models that knockout of the *Lrg1* gene, or its inhibition through a function-blocking antibody, delayed tumour growth and increased animal survival [86]. Moreover, in the absence or inhibition of LRG1, the tumour vasculature exhibited enhanced pericyte coverage and improved function suggesting that LRG1 prevents mature vessels from forming in the TME (Fig. 5). This may also explain the observed reduced vascular density as a stabilized vessel will be less responsive to the destabilizing cues required for sprouting angiogenesis. In support of LRG1 being a destabilizing factor in tumour vessels, *Lrg1* upregulation was also observed in brain tumours characterized by a profoundly altered and permeable blood–brain barrier [202].

In a recent landmark study, LRG1 has also been proposed as a major contributor to the metastatic niche, being synthesized by distant-organ endothelial cells in response to primary tumour-induced systemic inflammation (Fig. 3F). As a result, it plays a key role in conditioning the vascular bed for metastatic colonization, possibly by increasing the number of pro-metastatic perivascular cells [75]. This study also revealed that the contribution of LRG1 secreted by the vascular compartment is far more important than that synthesised by the liver, and that targeting LRG1 therapeutically may have utility in restricting metastatic cancer. Indeed, anti-LRG1 as a monotherapy offered a substantial survival advantage in a mouse model considered refractive to anti-VEGF therapy. Furthermore, the authors raise the intriguing notion that LRG1 may represent a novel angiocrine factor [203, 204]. A reduced dissemination of mouse melanoma cells was also observed in *Lrg1*^−/−^ mice further demonstrating a key role for LRG1 in tumour metastasis [90].

As indicated above, it is well-established that the vasculature of tumours is dysfunctional, being unstable, leaky, haemorrhagic, and poorly perfused. This is true not only for neovessels but also, in all likelihood, for co-opted vessels and implicates the presence of vasculopathic factors. Such abnormal tumour vessels not only thwart the delivery of therapeutics but also promote a more pro-oncogenic environment through establishing hypoxia and immunosuppression. This has led to the hypothesis that restoring vessel function, a process referred to as vessel or vascular *normalization*, will reverse the pro-oncogenic TME and improve the delivery and effectiveness of current standards of care and immunotherapies [205]. In our recent study we demonstrated that vascular normalization, brought about by LRG1 inhibition with a function-blocking antibody, enhanced the effectiveness of cytotoxic (cisplatin), adoptive T cell and checkpoint inhibitor therapies [86]. In the case of immunotherapies, there was a significant switch from a less inflamed TME to a more inflammatory, or “hot”, tumour response. Whether these beneficial outcomes are achieved by improved access, stabilization of vessels and reversal of endothelial anergy, altered vascular-immune cross talk or to a more general permissive immune *milieu* remains to be fully established. Nonetheless, while further studies are needed to elucidate the full range of activities LRG1 exerts during tumour progression, the evidence here reported firmly paves the way for LRG1 to become a novel and multifunctional target for the treatment of various cancer types.

### Neurological disorders

Under normal conditions, the central nervous system is not exposed to circulating levels of LRG1 because of the presence of the blood–brain barrier (BBB). Nevertheless, LRG1 has been implicated in the pathogenesis of several neurodegenerative diseases and proposed as a biomarker for the presence or progression of these conditions. Many neurodegenerative diseases are preceded by, or occur concurrently with, neuroinflammation and may also exhibit vascular disturbances.

Neuroinflammation increases with age and, interestingly, LRG1 levels in cerebrospinal fluid (CSF) in healthy subjects follow the same pattern [206]. Similarly, higher expression of LRG1 was observed, at the histological level, in brain sections of elderly patients, where LRG1 mainly localizes in the pericapillary area of astrocytic endfeet [207], possibly implicating age-related deterioration of BBB function resulting in diffusion of circulating LRG1 into the neural parenchyma. However, the greater prevalence of neurological disorders in the elderly cohort makes it unclear whether the increase in LRG1 production was caused by age, disease, or both. Indeed, some authors showed that the CSF concentration of LRG1 is significantly higher in patients with Parkinson’s disease, dementia, progressive supranuclear palsy, idiopathic normal pressure hydrocephalus and Alzheimer’s disease compared to healthy elderly controls [206, 208]. As indicated above, these data do not clarify the source of LRG1, the presence of which in the cerebral tissue may be due to local production or to increased permeability of the BBB. Nonetheless, the higher expression of LRG1 in the aged brain, and possible involvement in the pathogenesis of several neurodegenerative conditions, has been supported by animal studies [206]. Of note, the induction of cognitive impairment in mice by exposure to sevoflurane was shown to correlate with *Lrg1* expression in the hippocampus [209]. In line with this, while administration of selenomethionine, a well-known anti-oxidant compound, was reported to downregulate *Lrg1* in a mouse model of Alzheimer’s disease [210], transgenic mice overexpressing LRG1 in neurons and glial cells showed significant brain atrophy [206], corroborating the hypothesis that LRG1 contributes to brain inflammation and cognitive impairment.

LRG1 upregulation has also been described in idiopathic normal pressure hydrocephalus (INPH), a clinical syndrome of unknown aetiology, which leads to cognitive decline, gait disturbance and urinary symptoms [211, 212]. Raised levels of TGFβ and TGFβRII have been found in the CSF of patients with INPH [208]. Moreover, elevated TGFβ levels have been measured in the CSF of patients who developed hydrocephalus after subarachnoid haemorrhage. TGFβ released from platelets following subarachnoid haemorrhage might contribute to the development of communicating hydrocephalus by promoting fibrosis [213, 214]. In line with this observation, transgenic mice overexpressing TGFβ in the brain not only develop communicating hydrocephalus [215, 216] but also are characterized by increased deposition of extracellular matrix in the meninges, choroid plexus and other brain areas [216]. The exact role of LRG1 in the poorly defined pathophysiology of INPH is not known. However, considering its contribution to the fibrotic reactions mediated by TGFβ in other pathological settings, it is worthy of further investigation.

As with ischaemic events elsewhere in the body, alterations of LRG1 expression have been observed in cerebral infarctions, but data remain contradictory. For instance, while some authors have measured higher levels of LRG1 in the plasma of patients with stroke compared to healthy controls [217], others have reported the opposite in the same clinical condition [218]. Interestingly, increased LRG1 production was observed at the tissue level following middle cerebral artery occlusion (MCAO) in mice and rats [25, 218]. Meng *et al.* reported in this experimental model not only a significant TGFβ upregulation but also a positive correlation between LRG1 expression and microvessel density in the ischemic penumbra after cerebrovascular infarction [25]. Of note, penumbral neovascularization is known to be hyperpermeable, which is consistent with the well-established action of LRG1 in driving dysfunctional vessel growth. As observed in the heart, while LRG1 might play some beneficial functions on tissue revascularization following infarction, ectopic expression of LRG1 using an AAV vector exacerbated the ischemic/reperfusion injury caused by transient MCAO with regards to infarction volume and neurological score, which is in line with its highly pro-inflammatory nature [218]. In patients with supratentorial cerebral infarction, higher LRG1 serum levels correlate positively with infarction volume and stroke severity, suggesting that LRG1 might indeed worsen ischemia/reperfusion injury [218].

Finally, stressful events are known to affect the hippocampus, which has important functions for memory and learning [219], and increased *Lrg1* expression in this structure has been observed in animals subjected to chronic social stress [220] and recall of contextual fear memory [221], further validating the known link between stress and inflammation [222] and potential involvement of LRG1.

## Conclusions and future prospectives

In early studies LRG1 was described as an acute phase protein with a role in neutrophil differentiation and function, and these activities have largely stood the test of time. However, interest in this molecule has grown exponentially since its involvement in vascular disease was first reported in 2013. After almost a decade, a consistent flow of publications have described LRG1 as a multifunctional pro-inflammatory signalling molecule which is highly upregulated in many pathological settings. To date, most evidence suggests that LRG1 exerts its biological functions mainly by disrupting TGFβ signalling, although recent studies are beginning to elucidate additional pathways that may be involved (Fig. 6). Inherent differences in the inflammatory *milieu*, as well as in surface receptors and intracellular signalling molecules, might explain the dissimilarities observed among different cell types and support the hypothesis that LRG1 might disturb different signal transduction pathways in a highly cell-specific fashion.

The observation that *Lrg1*^*−/−*^ mice remain fertile and healthy over a normal lifespan reveals little about its function and suggests that LRG1 is not crucial but rather redundant in development and homeostasis. However, the fact that its structure has been highly conserved during evolution is consistent with the view that, despite being usually highly pathogenic, LRG1 presumably exerts certain beneficial functions. Indeed, several studies have appeared in recent years describing LRG1 as a key player in the organism’s acute response to injury and infections. In particular, LRG1 has been suggested to mediate the extravasation and activation of neutrophils, while enhancing a more general accumulation of immune cells at tissue level by neutralizing the cytotoxic effects of Cyt *c* and counteracting TGFβ anti-proliferative functions. Additionally, LRG1 favours wound closure by promoting the renewal of damaged epithelial cells and tissue vascularization, which is a fundamental requirement for a reparative inflammatory response. Notably, LRG1 is not involved in tissue remodelling during the physiological responses to injury but rather contributes by constraining TGFβ-induced ECM deposition therefore inhibiting possible fibrotic reactions. In view of the beneficial effects that LRG1 exerts on tissue repair, it has been suggested as a novel compound for the treatment of chronic wounds and heart failure, although animal studies are still required to corroborate these findings and to assess the safety and toxicity of such approaches. Whilst LRG1 is presumed to be necessary for the restoration of tissue homeostasis in these settings, it remains a pro-inflammatory cytokine and, as such, its function requires tight regulation to prevent pathogenic outcomes. Indeed, persistently high levels of LRG1 have been demonstrated to sustain the inflammatory response and contribute to disease progression through a broad range of biological functions (Fig. 5). Accordingly, the induction of high local levels of LRG1 in disease lesions contributes to the establishment of a highly inflamed, dysfunctional, and malignant microenvironment.

While affecting multiple cell types in a context-dependent manner, LRG1 can exert its angiopathic activity in virtually every pathological setting by disturbing endothelial cell function and the normal crosstalk between endothelial cells and pericytes. While this destabilizing function is necessary to prime vessels for sprouting, it also prevents the formation of a mature capillary network thus causing tissue malfunction. This, together with the observation that inhibiting LRG1 function attenuates disease progression through restoration of vascular homeostasis, has paved the way for the development of a humanized anti-LRG1 antibody that may be tested clinically in the near future. In particular, Magacizumab is an IgG4 antibody specifically designed to minimize the risk of inflammatory reactions and whose efficacy in limiting vascular leakage and tumour progression has been already demonstrated in murine models of nvAMD [5] and melanoma [6], respectively. These encouraging results were followed by the development of an anti-LRG1 Fab fragment exhibiting even higher therapeutic benefits, especially in the context of ocular injections where the lack of the Fc fragment, and the reduced molecular weight, lend themselves to intraocular delivery [5]. As LRG1 can affect both existing and newly formed vessels, it is tempting to speculate that counteracting LRG1 angiopathic activity might be beneficial in a wide range of diseases, ranging from cancer to the vascular complications associated with inflammation and diabetes. Considering the surge in the number of overweight and obese individuals, as well as a rising aging population, there is an urgent need for more effective and targeted interventions, and we propose tackling LRG1 function as a promising strategy. In fact, compared to other therapeutic targets associated with abnormal angiogenesis, such as VEGF, LRG1 exhibits multiple advantages. Firstly, while rescuing the phenotype of diseased vessels, LRG1 blockade may allow amelioration of the pathogenic microenvironment simultaneously at multiple levels, for instance by providing additional anti-inflammatory benefits and limiting excessive fibrosis. This is particularly relevant as neovascular and fibrotic responses often go hand in hand in late-stage diabetes and chronic inflammation. As an upstream modifier of TGFβ signalling, LRG1 exerts its functions by redirecting the binding of TGFβ to its various receptors in a highly cell-dependent fashion. However, since LRG1 is highly expressed exclusively in disease, its blockade may be expected to interfere selectively with the pathological effects of TGFβ signalling without impacting unduly on its many essential physiological functions.

Not only does LRG1 represent an interesting therapeutic target, but it also stands as a potentially important biomarker. In response to various inflammatory stimuli, including injury, infections, autoimmune conditions, and tumour growth, both liver and tissue lesions contribute to LRG1 blood levels. Indeed, increased circulating levels of LRG1 contribute to multi-protein biomarker signatures for the diagnosis and prognosis of a broad plethora of human diseases. Nevertheless, whether, and to what extent, LRG1 sourced by the liver also participates in disease progression remains to be clarified.

To summarize, LRG1 is a pleiotropic acute phase-like protein produced as part of the organism’s first line of defence. However, high levels of LRG1 are highly pathogenic and further contribute to tissue damage. Therefore, taking into consideration the crucial role of LRG1 as a contributing factor in many pathological settings, we propose the translation into the clinic of a function-blocking antibody as a therapeutic option for a wide range of conditions.

## Data Availability

The original data presented in this study (Fig. 3) are available from the corresponding author on reasonable request.

## Abbreviations

AGE: Advanced glycation end
ALK 1/5: Activin receptor-like kinase 1/5
Apaf-1: Apoptotic peptidase activating
APP: Acute phase protein
BBB: Blood–brain barrier
BMI: Body mass indes
BMP: Bone morphogenic protein
BPD: Bronchopulmonary dysplasia
CAF: Cancer-associated fibroblast
COPD: Chronic obstructive pulmonary disease
CRC: Colorectal cancer
CRP: C reactive protein
CSF: Cerebrospinal fluid
Cyt * c*: Cytochrome C
DKD: Diabetic kidney disease
ECM: Extracellular matrix
EGF(R): Epidermal growth factor (receptor)
EMT: Epithelial-mesenchymal transition
ENG: Endoglin
G-CSF: Granulocyte-colony stimulating factor
HIF1α: Hypoxia-inducible factor 1α
HFDα: High fat diet
ICAM-1: Intercellular adhesion molecule 1
IL-6(R): Interleukin-6 (receptor)
INHP: Idiopathic normal pressure hydrocephalus
IRS: Insulin receptor substrate
LPS: Lipopolysaccharide
LRC: Leucin-rich C-terminal domain
LRG1: Leucine-rich α-2 glycoprotein 1
LRR: Leucine-rich repeat
miRNA: MicroRNAs
MCAO: Middle cerebral artery occlusion
MLC: Myosin light chain
NET: Neutrophil extracellular trap
nvAMD: Neovascular age-related macular degeneration
OA: Osteoarthritis
PDGF: Platelet-derived growth factor
PDR: Proliferative diabetic retinopathy
RA: Rheumatoid arthritis
RAS: Renin-angiotensin system
RUNX1: Runt-related transcription factor 1
SAA: Serum amyloid A
SARS: Severe acute respiratory syndrome
SREBP1: Sterol regulatory-element binding protein 1
STZ: Streptozotocin
T1 or T2DM: Type 1 or type 2 diabetes mellitus
TGFβ(RII): Transforming growth factor β (receptor II)
UC: Ulcerative colitis
TNFα: Tumour necrosis factor
VCAM-1: Vascular cell adhesion molecule 1
VEGF(R): Vascular endothelial growth factor (receptor)
